# Chirality Transfer, Memory and Sensing Activated by a Supramolecular Chiral Auxiliary Approach in Nanostructured, Tautomerically Prochiral Triptycene-Fused Benzimidazoles

**DOI:** 10.1021/jacs.6c03689

**Published:** 2026-05-07

**Authors:** Giovanni Preda, Riccardo Mobili, Giovanna Longhi, Massimiliano Meli, Giorgio Colombo, Valeria Amendola, Dario Pasini

**Affiliations:** † Department of Chemistry and INSTM Research Unit, 19001 University of Pavia, Via Taramelli 12, 27100 Pavia, Italy; ∥ Dipartimento di Medicina Molecolare e Traslazionale, University of Brescia, Viale Europa 11, 25123 Brescia, Italy; ‡ INO−CNR, Research Unit Brescia, c/o CSMT via Branze, 45, 25123 Brescia, Italy; § Istituto di Scienze e Tecnologie Chimiche “Giulio Natta” − “SCITEC” CNR, Via Mario Bianco 9, 20131 Milano, Italy

## Abstract

We report the synthesis and characterization of stimuli responsive, adaptive organic chiral nanoparticles assembled using a “stereochemically fluid”, triptycene-fused benzimidazole, which is chiroptically activated by the noncovalent interaction with enantiopure tartaric acid, acting as a “supramolecular chiral auxiliary”. Under appropriate experimental conditions, the formation of chiral supramolecular aggregates exhibits remarkable chiroptical properties (electronic circular dichroism, ECD, and circularly polarized luminescence, CPL) with the possibility of addressing their controlled manipulation, reversion between the states, and chirality memory properties. Furthermore, the nanostructured system is capable of selective sensing of the Cu^2+^ ion through the modulation of its chiroptical properties. In this manner, we provide an unprecedented way to introduce chirality onto the triptycene skeleton to assemble well-defined chiral nanoparticles with dimensions of several hundred nanometers and to reversibly store chiral information and activate chiroptical sensing properties.

## Graphical abstract

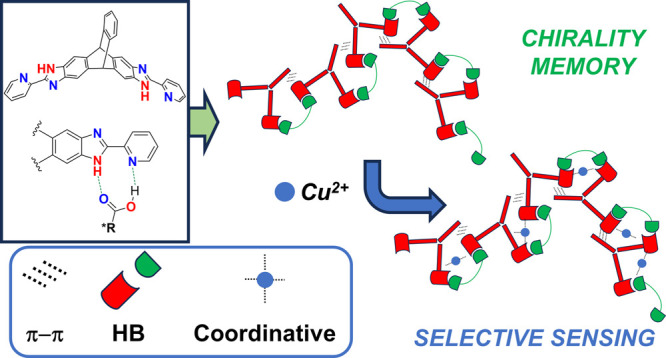

## Introduction

Supramolecular interactions are intimately connected with the most important living functions, such as replication, storage, transfer, and amplification of biological information, with a delicate equilibrium between molecular and supramolecular chirality in charge of such processes.^1,2^ The bottom-up control of supramolecular chirality has emerged in recent decades as an important field of research covering numerous technological areas: sensing,^3–5^ self-assembly of nanomaterials,^6^ asymmetric synthesis,^7,8^ artificial biomimetic systems,^9–11^ optics,^12,13^ and information storage.^14^

Emergence of supramolecular chirality can be triggered by chirality transfer: an element of molecular chirality (chiral constituent), point, axial, planar, or helical, can induce chiroptical properties (electronic circular dichroism, ECD, vibrational circular dichroism VCD, and circularly polarized luminescence, CPL) in a nonchiral chromophoric constituent via supramolecular assembly. Such induction can occur through hydrogen bond, π–π stacking, coordinative, solvophobic, or electrostatic interactions, and such multicomponent systems can be extremely complicated, since scenarios such as self-sorting or coassembly may take place.^15,16^ Obtaining, studying, and manipulating chiral multicomponent systems to achieve smart materials capable of expressing sophisticated chiroptical properties are major goals of modern supramolecular chemistry.

Imidazoles are an essential part of biomolecules^17^ in which two tautomeric forms are present because the hydrogen can be bound to one or the other nitrogen atom; their benzoannulated analogues, possessing essentially identical properties, have gathered increasing attention regarding their implementation in self-assembled supramolecular structures.^18–20^ The examples reported so far are all based on the use of planar benzimidazole chromophores.^21–23^ Triptycene is a nonplanar π-conjugated synthon, which has fascinated generations of chemists, and has already been established in materials and supramolecular chemistry thanks to its versatility and robustness.^24,25^ The main possibility for inducing chirality on it is usually achieved through covalent functionalization following chiral substitution patterns.^26,27^ Due to its extremely limited conformational freedom, it is widely regarded as an organic system in which chirality is expressed in an exceptionally robust fashion. To our knowledge, no one has ever designed a “stereolabile” or “chirally adaptive” triptycene in which stereochemistry can be easily switched dynamically between different isomers through pure noncovalent interactions. We recently reported the synthesis and characterization of a small library of triptycene-fused benzimidazoles,^28^ among which is compound **1a**; see Figure 1A.

**Figure 1:**
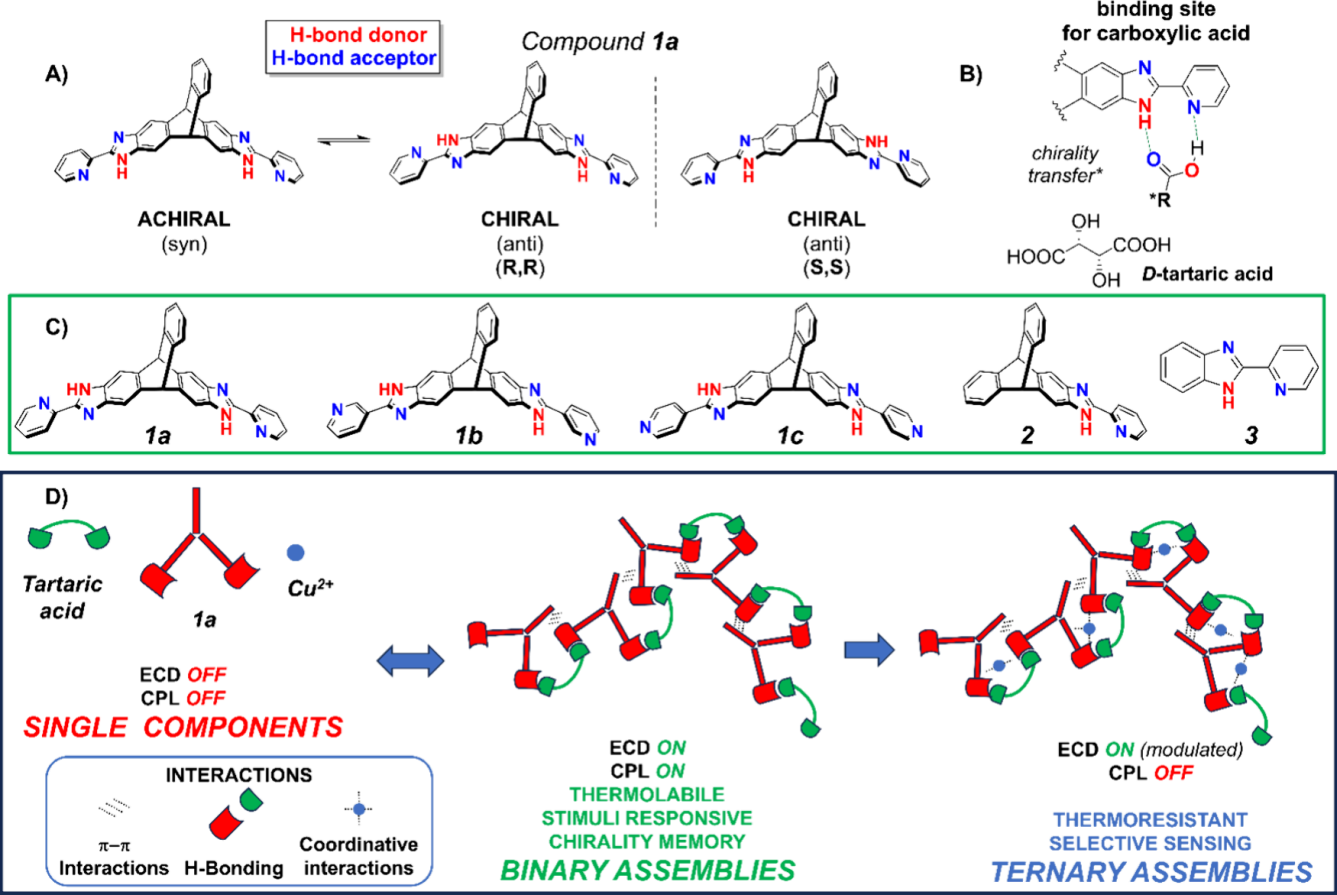
(A) Tautomeric equilibrium of triptycene-fused benzimidazole **1a**. (B) Possible noncovalent interaction mechanisms of compound **1a** with chiral carboxylic acids via hydrogen bonds. (C) Benzimidazoles and chiral carboxylic acids used in this work. (D) Chiral aggregates composed of **1a**, enantiopure tartaric acid (**TA**), and Cu^2+^.

We here report the use of the “stereochemically fluid” triptycene-fused benzimidazole **1a**, which, thanks to the noncovalent interaction with enantiopure tartaric acid (**TA**) (Figure 1B), under appropriate experimental conditions leads to the formation of chiral nanostructured aggregates that exhibit remarkable chiroptical properties (ECD and CPL) with the possibility of addressing the controlled manipulation, reversion between the states, and chirality memory properties.

Furthermore, the nanostructured system is capable of selective sensing of the Cu^2+^ ion through modulation of its chiroptical properties. The mechanism of assembly and sensing has been elucidated by comparison with model compounds (Figure 1C): compounds **1b** and **1c**, nonplanar triptycene derivatives possessing two benzimidazole moieties and with the same prototropic tautomerism as in **1a**, triptycene derivative **2**, and the planar analogue **3**, possessing a single benzimidazole moiety. Molecular dynamics has aided the rationalization of the early stages of the assembly process.

The illustration of the interconversion/control among the different aggregation modes of **1a**/**TA** assemblies, which are held together by a combination of π–π interactions, hydrogen bonding, and coordinative interactions, is reported in Figure 1D.

## Results and Discussion

### Supramolecular Synthesis

The shape-persistent, nonplanar π-conjugated triptycene backbone is “stereochemically adaptive”, and compound **1a** can be referred to as “pro-tautomerically chiral” (Figure 1A). The rotation of the pyridine around the aryl–aryl bond is fast, and as a consequence of the exchange of the NH proton between the two nitrogen atoms of the imidazole moiety, **1a** can occur as an achiral tautomer in which the benzimidazole hydrogens are on the same side (“syn”) or as two distinct nonsuperimposable enantiomers in which the hydrogens are on opposite sides (“anti”). It is known that the benzimidazole functional groups can form complexes with carboxylic acids via hydrogen bonding,^29,30^ so that, we reasoned, in the presence of a chiral carboxylic acid as a “supramolecular chiral auxiliary”, the chiral information could be “imprinted” by selecting one of the possible stereoisomers and incorporating them into a chiral supramolecular structure, endowed with chiroptical properties. **TA**, a naturally available chiral dicarboxylic acid belonging to the “chiral pool”, is potentially capable of establishing specific and complementary hydrogen bonds with **1a**, as depicted in Figure 1B. The insolubility of the two components in noncompeting, apolar solvents did not allow the characterization of the hydrogen bonding patterns using NMR spectroscopy.

Triptycene-fused benzimidazoles are insoluble in water while they are soluble in organic solvents such as DMSO and, to a lesser extent, THF, MeOH, and MeCN. In contrast, **TA** is perfectly soluble in both water and the above-mentioned organic solvents. We carried out extensive preliminary experiments in which we varied the concentration of **1a**, **TA** equivalents, solvent (DMSO)/antisolvent (H_2_O) ratio, and aging time. In a typical experiment, the supramolecular aggregates were prepared from concentrated stock solutions of **1a** and enantiopure **TA** in DMSO using different amounts of solvent (DMSO)/antisolvent (H_2_O) in well-defined ratios and *ca*. 5 × 10^–5^ M concentration of **1a**. The stable colloidal dispersion was kept in a refrigerator (4 °C) for 7 days. Under such conditions, we were able to optimize the chiroptical response and obtain an effective transfer of the nonchromophoric stereochemical information contained in **TA** to the triptycene-fused benzimidazole **1a**, triggering an intense Cotton effect measurable in the spectral range (300–450 nm) associated with the benzimidazole chromophore (Figure 2A).

**Figure 2:**
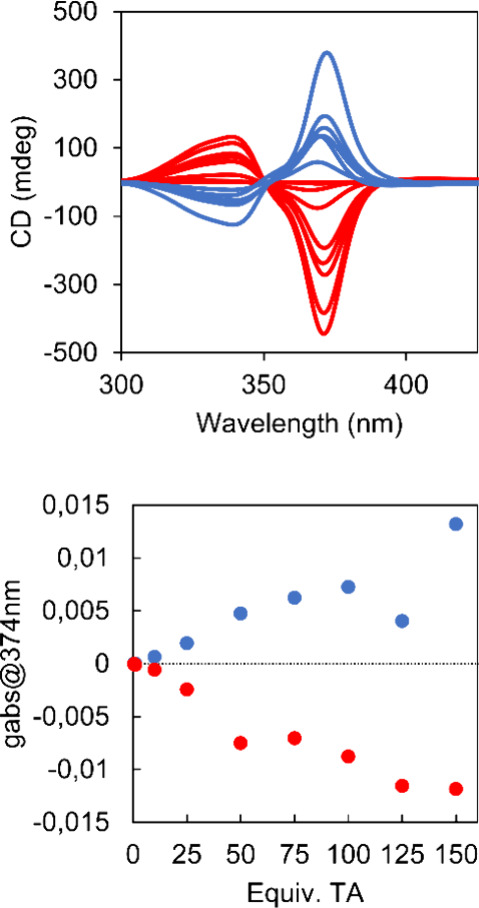
ECD spectra (top) of **1a**/***D***-**TA** (blue) and **1a**/***L***-**TA** (red) aggregates with ratios from 1/1 to 1/150 equiv of **TA** in 15% (v/v) DMSO/H_2_O and (bottom) trend of *g*_abs_ at 374 nm versus equiv of **TA**.

### Spectroscopical Characterization of the Aggregates

The spectra of aggregates formed by **1a** and ***L***- or ***D*** **-TA** in a 15% (v/v) DMSO/H_2_O solvent mixture show the appearance of a very strong Cotton bisignate effect with anisotropy factor (*g*_abs_) values up to 1.3 × 10^–2^ for ***D***-**TA** and −1.2 × 10^–2^ for ***L***-**TA** at the wavelengths (300–450 nm) attributable to the electronic transitions of the benzimidazole chromophore (Figure 2A). The *g*_abs_ values are among the highest for self-assembled organic structures.^31^ The CD signal started to emerge with small equiv of **TA** (<5 equiv), but we observed an enhancement employing a large excess of **TA** (maximum signal for equiv of **TA** *ca*. 100–150). The respective UV/vis spectra (Figure S1) showed little variation of the spectral shape and of the λ_max_ upon increasing additions of **TA**.

Importantly, these assemblies show good reproducibility and stability over time: once kept at 4 °C, the CD measurements could be replicated and were superimposable after more than one year. Furthermore, they are operative (albeit with slightly lower *g*_abs_) with other solvent mixtures tested such as THF/H_2_O (Figure S2A).

Intrigued by these encouraging preliminary results, we began to investigate in detail the factors that govern the multicomponent self-assembly and symmetry breaking in the aggregated state. The solvent/antisolvent ratio was found to be a crucial parameter capable of regulating the supramolecular self-assembly. To investigate this effect, we prepared different mixtures, keeping the concentration and **1a**/***L***-**TA** molar ratio constant (1/75) and varying the solvent/antisolvent composition. By recording ECD spectra of these mixtures, it was possible to derive the optimal cosolvent composition (15% DMSO/H_2_O, Figure 3 or 11% (v/v) THF/H_2_O, Figure S2B) for maximizing the chiroptical response.

**Figure 3:**
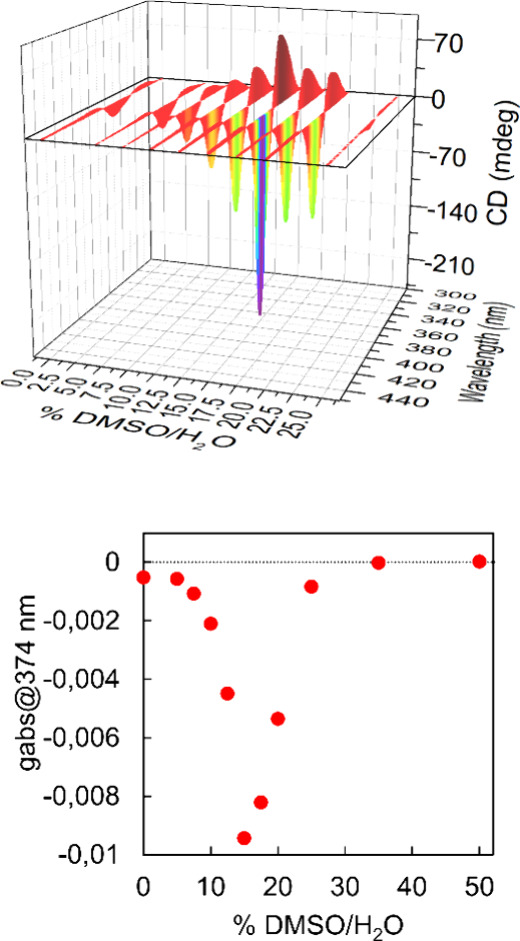
3D and 2D plots of ECD spectra (top) of **1a**/***L***-**TA** 1/75 aggregates in different (v/v) DMSO/H_2_O solvent mixtures and (bottom) trend for *g*_abs_ (374 nm) as a function of (v/v) DMSO/H_2_O.

As shown in Figure 3, the evolution of chirality transfer from ***L***-**TA** to triptycene-fused benzimidazole **1a** is not trivial: it is operative at even 0% DMSO; it increases dramatically from 0 to 15% DMSO (maximum ECD signal) and finally disappears completely for relative quantities of DMSO greater than 25%. The assembly can also be partially probed by UV–vis: for DMSO percentages lower than 17.5%, the appearance of a shoulder at *ca*. 370 nm can be noted, attributable to J-aggregates held together by π–π and hydrogen bond interactions (Figure S3A). At DMSO percentages greater than 20%, this band decreases and shifts toward smaller wavelengths (345 nm), ascribed to the molecularly dispersed triptycene-fused benzimidazole chromophores dissolved in the solvent that are no longer able to effectively interact with ***L***-**TA**. Such coassembly behavior is indicative of the delicate interplay between the synergistic interactions among individual components (H-donors, H-acceptors, J-aggregation) and between components and solvent. At low percentages of DMSO, the components, although partially assembled, are “rigid”, because of their poor solubility, which prevents “trial-and-error” movements required to achieve efficient synergistic coassembly. On the other hand, at optimal percentages of DMSO, thanks to enhanced solubility, they possess the optimal “flexibility”, while retaining the necessary intercomponent interactions capable of driving chiral assembly. Finally, at high percentages of DMSO, due to complete solubilization of the components, solvent/component interactions overwhelm supramolecular interactions between the components, completely precluding coassembly. Similar remarks can be made for THF/H_2_O mixtures, exhibiting maximum ECD response at *ca*. 11% (v/v) THF/H_2_O (Figures S2B and S3B).

To elucidate the key structural features of benzimidazoles that may affect the assembly, we examined two other isomers of **1a**: **1b** (*meta*-pyridine) and **1c** (*ortho*-pyridine). Under the same conditions, i.e., 15% (v/v) DMSO/H_2_O and a large excess of **TA**, **1b** (Figure S4) is able to exhibit a small ECD signal (*g*_abs_ less than 9 × 10^–4^), while **1c** (Figure S5) turned out to be completely inactive. This is probably due to the preorganization and stronger ability of the *ortho*-pyridine/benzimidazole fragment to “chelate” via hydrogen bonding **TA** (Figure 1B). In contrast, these multiple interactions with **TA** are limited (**1b**) or completely prevented (**1c**). To separate the importance of the shape-persistent, tautomerically “chiral” triptycene bicyclic skeleton from the benzimidazole chromophore itself, we tested, under the same conditions, the nonplanar and planar monobenzimidazole analogues **2** and **3** (Figure S5). Both were unable to show ECD activity, suggesting the importance of the tautomerically “fluid” nonplanar skeleton of triptycene during the processes of chirality transfer and symmetry breaking in the aggregated state.

The presence of supramolecular aggregates **1a**/***L***-**TA** with size of approximately 460 nm was detected via dynamic light scattering (DLS) measurements. These aggregates are essentially absent in v/v DMSO/H_2_O mixtures surpassing 30%. By recording variable-temperature measurements (VT-DLS), it was possible to follow the “melting” of structures into smaller fragments. In the 25–70 °C temperature range, sizes show only minor variations; however, at 90 °C, binary coassemblies composed of **1a**/***L***-**TA** break up into smaller fragments with size *ca*. 25 nm (Figure 4 and Figure S6). These observations agree with the VT-ECD experiments that will be described later. Using DLS, it was also possible to observe the presence of the aggregates composed only of **1a** in 15% (v/v) DMSO/H_2_O with a size of approximately 700 nm (Figure 4).

**Figure 4:**
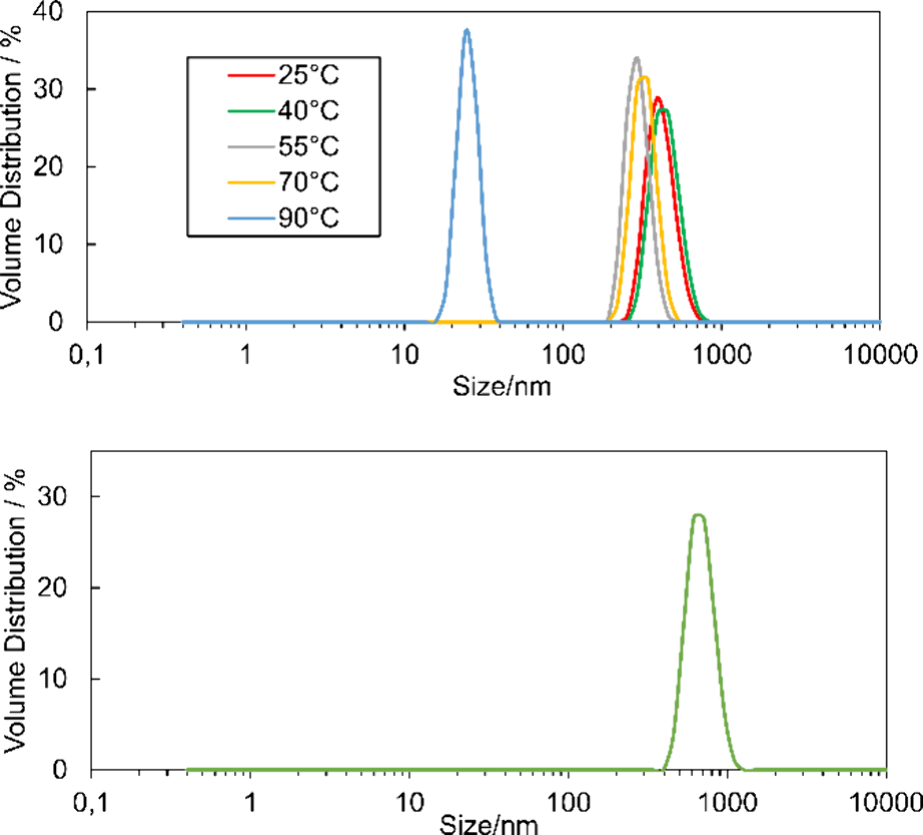
VT-DLS measurements (top) from 25 to 90 °C of the aggregates **1a**/***L***-**TA** 1/125 in 15% (v/v) DMSO/H_2_O. DLS of a colloidal suspension (bottom) of **1a** (5.2·x 10^–5^ M) in 15% (v/v) DMSO/H_2_O.

Not surprisingly, **1a** itself can form self-assembled random aggregates via hydrogen bonding and J-aggregation, which can become chiral in the presence of ***L***- or ***D***-**TA**. Indeed, the “inertia” toward **TA** incorporation, also evidenced by the time needed (at least 1 week) for effective assembly, may explain why it is necessary to use a large excess of **TA**, making use of the law of mass action, to push the supramolecular system to reassemble, trigger, and maximize the intense ECD signal.^32^

Insights into the structure of the aggregates can also be inferred using fluorescence emission spectroscopy. Molecularly dispersed **1a** in DMSO exhibits an absorption maximum at λ_max_ = 355 nm with an emission peak at λ_max_ = 389 nm. The modest stokes-shift (34 nm) is attributable to the molecular rigidity of the chromophore, which is typical for small π-conjugated organic molecules (Figure S7). In 15% (v/v) DMSO/H_2_O, the shift in the maximum absorption peak (λ_max_ = 370 nm) can be explained with the formation of intermolecular interactions in the random supramolecular J-aggregates. The emission (λ_max_ = 517 nm, stokes-shift 147 nm) is drastically changed, a situation typical of the formation of metastable J-aggregates, as previously reported.^33^ Upon the presence of an excess of **TA**, we observed the blue-shift of maximum absorption (λ_max_ = 366 nm) and emission (λ_max_ = 478 nm), confirming the already described evolution into “chiral” coassemblies. Interestingly, the absorption λ_max_ does not revert back to the value in which molecular components are completely dissolved (λ_max_ = 355 nm), indicating that J-type or π–π interactions are important in the stabilization of the overall supramolecular chiral nanostructures: in other words, **TA** coordination does not distance benzimidazole molecules too much further apart to suppress J-aggregation.

The chiral **1a**/**TA** aggregates exhibited circularly polarized luminescence (CPL) properties with anisotropy factors in the order of 2 × 10^–3^ (Figure 5). The CPL activity correlates with the CD activity; that is, optical activity shows up both in absorption and in emission when supramolecular chirally ordered aggregates are formed, whereas the random **1a** aggregates, as expected, do not show CD or CPL activity. Besides, the observed CPL sign is the same as the first CD-band sign, calling for similar configurational properties of the ground and excited states giving rise to the observed chiral signal in absorption and emission, respectively: here, it is the supramolecular structure dictating the geometrical properties of both states and thus the chiral response of the ground and excited states, similarly to previous reports in the literature about supramolecular chiral assemblies.^34,35^

**Figure 5:**
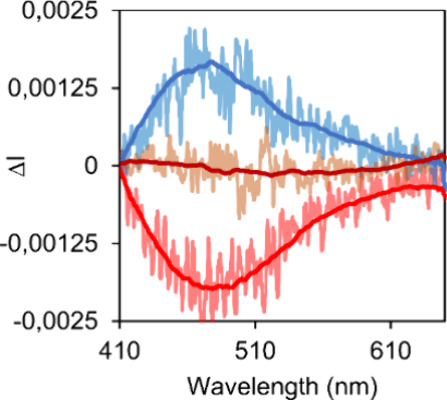
CPL spectra of **1a**/***D***-**TA**, **1a**/***L***-**TA**, and **1a** assemblies in 15% (v/v) DMSO/H_2_O.

### Stimuli-Responsive Assembly/Disassembly and Chirality Memory Properties

To test the capabilities of aggregates to act as stimuli-responsive chiroptical smart materials, we started to explore methods for the controlled manipulation of chiroptical properties. Variable temperature ECD (VT-ECD) spectra were initially recorded. Upon heating from 20 to 90 °C, we noticed the disappearance of the ECD signal coupled with the blue shift of the UV–vis band at 366 nm, attributable to the chiral aggregates toward individual components (Figure 6).

**Figure 6:**
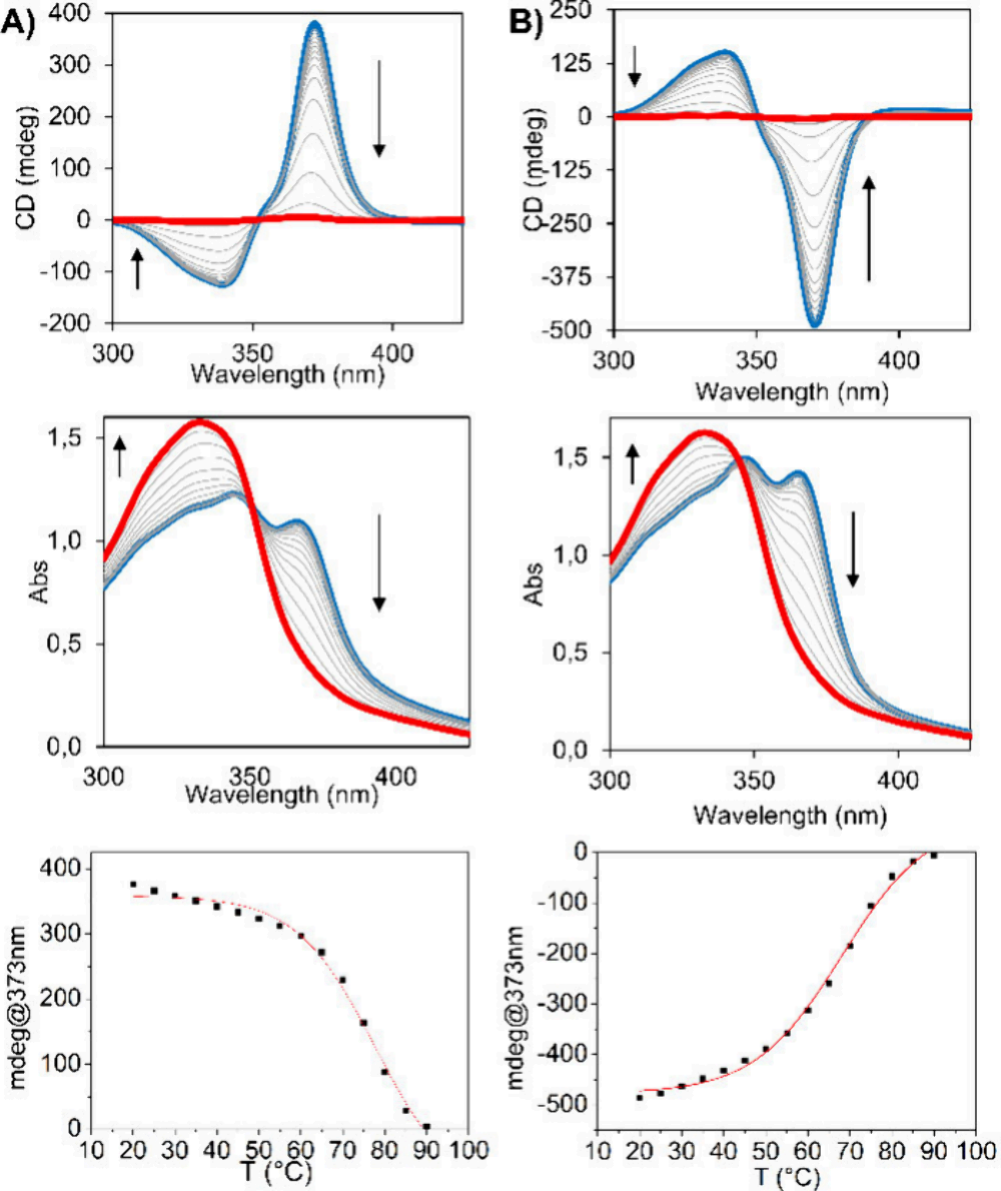
(A) VT-ECD (top) and VT-UV–vis (middle) spectra from 20 °C (blue line) to 90 °C (red line) of **1a**/***D***-**TA** assemblies in 15% (v/v) DMSO/H_2_O; bottom: sigmoidal variation of ECD signal at 373 nm as a function of temperature. (B) VT-ECD (top) and VT-UV–vis (middle) spectra (from 20 to 90 °C) of **1a**/***L***-**TA** assemblies in 15% (v/v) DMSO/H_2_O; bottom: sigmoidal variation of ECD signal at 373 nm as a function of temperature.

Clearly, high temperatures can “disassemble” chiral aggregates into individual components, thus erasing ECD activity. Indeed, the same analysis performed on solely **1a** in the same solvent mixture (15% (v/v) DMSO/H_2_O) showed the blue shift of the UV–vis band at 370 nm, corresponding to the achiral J-aggregates, toward the λ_max_ of the dissolved and disassembled molecular **1a**. Indeed, no associated ECD activity is detected throughout the VT experiments (Figure S8).

The isosdesmic-like transition from chiral superstructure to individual molecular components can be followed via ECD spectroscopy.^36^ Inspired by biochemical/biological studies involving stability of naturally occurring suprastructures,^37–39^ we used data gained from these analyses as descriptors of thermal stability. The VT-ECD denaturation curves were fitted with a Boltzmann sigmoidal function from which it was possible to derive the melting temperature (*T*_m_) data of the superstructures depicting the thermal stability of the aggregates (*T*_m_ = *ca*. 68 and 77 °C for ***L***-**TA** and ***D***-**TA** respectively, Figures S9–S12). Once the heating was completed, the solutions were allowed to rest at 4 °C for at least 1 week, after which another VT-ECD round was carried out. Interestingly, the aggregates displayed nonobvious self-recovery abilities. After the aging time, chiroptical activity can be recovered, even with declining efficiency for ***L***-**TA** and ***D***-**TA** after the first and second rounds of VT-ECD for both enantiomeric aggregates (Figure 7). To our knowledge, this is one of the few examples of multicomponent chiroptical materials capable of self-recovery with good efficiency and is due to the soft noncovalent interactions operative between the two components (hydrogen bonds and J-aggregation).^40–43^

**Figure 7:**
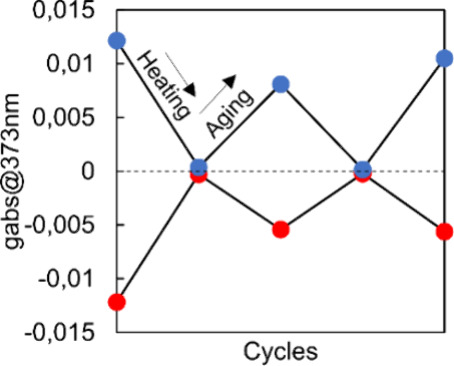
*g*_abs_ trend of binary aggregates upon different cycles of VT-ECD experiments.

To test the influence of hydrogen bonding on the assemblies, we performed ECD spectra at different pH values. In our expectations, by changing the pH (very basic or very acidic pH), the hydrogen bonds responsible for self-assembly should be affected, eventually disrupting the chiral assemblies and erasing the ECD activity. Surprisingly, the aggregates instead showed chiral memory properties imprinted by a supramolecular chiral auxiliary.

After measuring the native pH of the aggregates (2.7), they were basified by adding ca. 400 equiv of a 0.75 M NaOH solution, raising the pH up to 11.5. Under these conditions, ***L***-**TA** is fully converted into dicarboxylate anion, which is virtually unable to interact via acid–base interactions and hydrogen bonding with **1a**. However, the chiroptical activity remained essentially unchanged, even after more than six months after basification (Figure 8A). Similarly, acidification of the aggregates with a 1 M solution of trifluoroacetic acid (TFA, *ca*. 400 equiv) to pH 1.7 did not result in appreciable alterations of chiroptical activity within six months. This implies that the large excess of TFA (strong acid) is unable to compete with ***L***-**TA** (weak acid) in protonating **1a**, which should lead to a rupture of the chiral supramolecular aggregates and to a loss of ECD activity.

**Figure 8:**
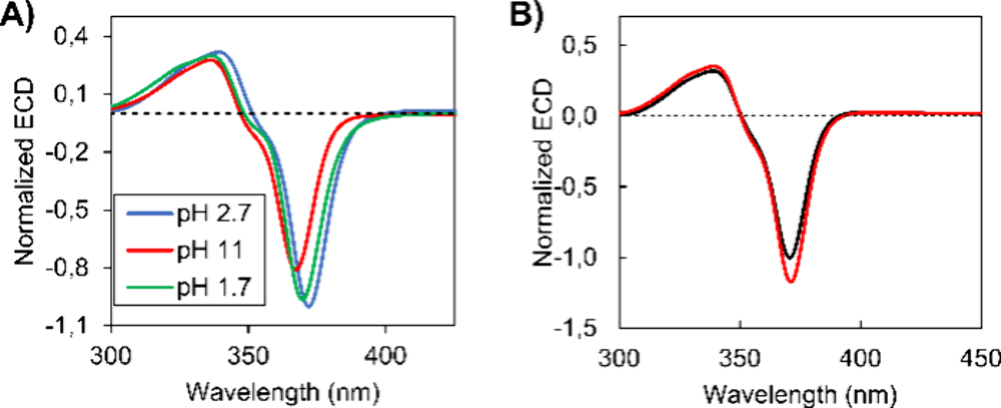
(A) ECD spectra of **1a**/***L***-**TA** 1/125 assemblies in a 15% DMSO/H_2_O mixture at different pHs obtained by addition of concentrated solution of NaOH or TFA. (B) ECD spectra of **1a**/***L***-**TA** 1/125 aggregates in a 15% DMSO/H_2_O mixture before (black line) and after (red line) the addition of 125 equiv of ***D***-**TA**.

The surprising tolerance to chemical stimuli such as large pH variations points toward chiral memory properties of these J-aggregates once they are formed. To further confirm memory properties of aggregates, we added 125 equiv of the opposite enantiomer (***D***-**TA**) to an already assembled suspension of **1a**/***L***-**TA** 1/125 aggregates. In this way, we removed the stereochemical information initially present, obtaining a racemate with 0% ee in **TA**. After more than 1 month after addition, however, the ECD activity was practically unaffected despite the final ee of 0% (Figure 8B and Figure S13).

### Assembly of Ternary Aggregates and Sensing of Metal Ions

Given that **1a** has many potential sites for interaction with metal ions, we tested the ability of **1a**/***L***-**TA** coassemblies to serve as chiroptical sensors^44–47^ for water-soluble metal cations. Concentrated solutions of metals (2 equiv of Zn^2+^, Cu^2+^, Ni^2+^, Co^2+^, Ba^2+^, and Eu^3+^ vs **1a**) were added to the assembled aggregates of **1a**/***L***-**TA** (Figure 9), as preliminary studies have shown that the excess of metals before the formation of the aggregates reduces or completely prevents the formation of chiral assemblies (Figure S14). This is probably because the initial presence of metals saturates the benzimidazole *o*-pyridine chelating sites, preventing chiral assembly with **TA**.

**Figure 9:**
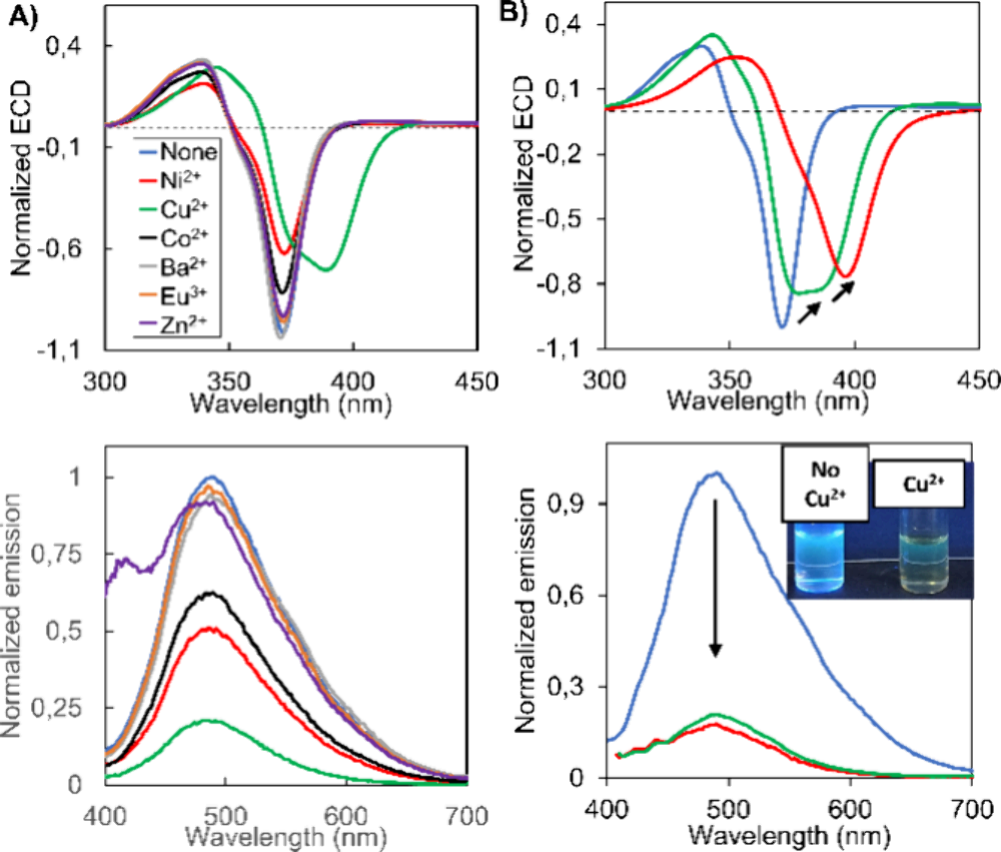
(A) ECD (top) and fluorescence emission (bottom) spectra of **1a**/***L***-**TA** 1/125 aggregates in 15% (v/v) DMSO/H_2_O in the presence of 2 equiv of different metal ions. (B) ECD (top) and fluorescence emission spectra (bottom) of **1a**/***L***-**TA** 1/125 aggregates in DMSO/H_2_O in the presence of different amounts of Cu^2+^: 0 (blue), 25 (green), 100 (red) equiv. Bottom inset: photo of **1a**/***L***-**TA** binary aggregates in the absence and the presence of 2 equiv of Cu^2+^ under a 365 nm TLC lamp.

The ECD and UV–vis spectra (normalized with respect to the corresponding spectrum recorded before the addition of metal) do not show important changes for Zn^2+^, Ba^2+^, and Eu^3+^, while for Ni^2+^ and Co^2+^, a slight disassembling tendency is detected (Figures S16–S21). Remarkably, 2 equiv of Cu^2+^ can considerably alter the ECD spectrum with a broadening and red shift of the ECD band from 366 to 400 nm (Figure S15A). The band shift is complete after addition of a large excess of Cu^2+^ (100 equiv). In the UV–vis spectrum, we also observed the red shift of the J-aggregate band from 370 to 400 nm. Interestingly, Cu^2+^ can selectively quench (5×) the fluorescence of the aggregates with respect to the initial value. Although less efficient, Ni^2+^ (2×) and Co^2+^ (1.7×) can compete with Cu^2+^ for fluorescence quenching, while all other metals tested do not impact the emissive properties (Figure 9). Since emission is quenched when Cu^2+^ is present, it becomes impossible to record any CPL signal (CPL off), thus potentially making this system one of the few examples of a CPL sensor.^48,49^

The aggregates incorporate Cu^2+^ thanks to the presence of numerous imidazole and pyridine nitrogen that can establish coordinative interactions with metal ions, thus forming a tricomponent **1a**/***L***-**TA**/**Cu** ^**2+**^ assembly held together by a combination of hydrogen bonding, J-aggregation, and coordinative interactions.

The proximity of the metal ion to the benzimidazole chromophore quenches its fluorescence, probably by a photoinduced electron transfer mechanism. Therefore, the aggregates can be considered as UV–vis, ECD, and fluorescent (chiro)­optical sensors toward Cu^2+^.

We found that the presence of Cu^2+^ can radically change the thermal stability of the aggregates: VT-ECD experiments in the presence of excess Cu^2+^ showed that the ECD signal decreases but continues to persist even at high temperatures (90 °C). The change of ECD signal vs *T* (°C) is strongly modified (Figure 10) with the disappearance of the sigmoidal character found in **1a**/***L***-**TA** (Figure 6) binary aggregates. The original chiroptical activity is nearly completely recovered ca. 20 min after heating, and the recovery is complete after a few hours (Figure S22), suggesting that the structures remain essentially unaffected by heating and the change in ECD activity is probably due to molecular motions activated by high temperatures. In fact, in the case of **1a**/***L***-**TA** binary assemblies, upon VT-ECD experiments, the chiroptical activity was only partially recovered after at least 1 week of the aging period (Figure 6 and Figure 7). These observations indicate how the stability of **1a**/***L***-**TA**/**Cu** ^**2+**^ ternary aggregates is drastically increased in comparison to **1a**/***L***-**TA** binary aggregates in the investigated temperature range and provide a suitable method to transform the reversibly thermolabile chiroptical activity of **1a**/***L***-**TA** binary aggregates into thermoresistant **1a**/***L*** **-TA**/**Cu** ^**2+**^ tricomponent aggregates.

**Figure 10:**
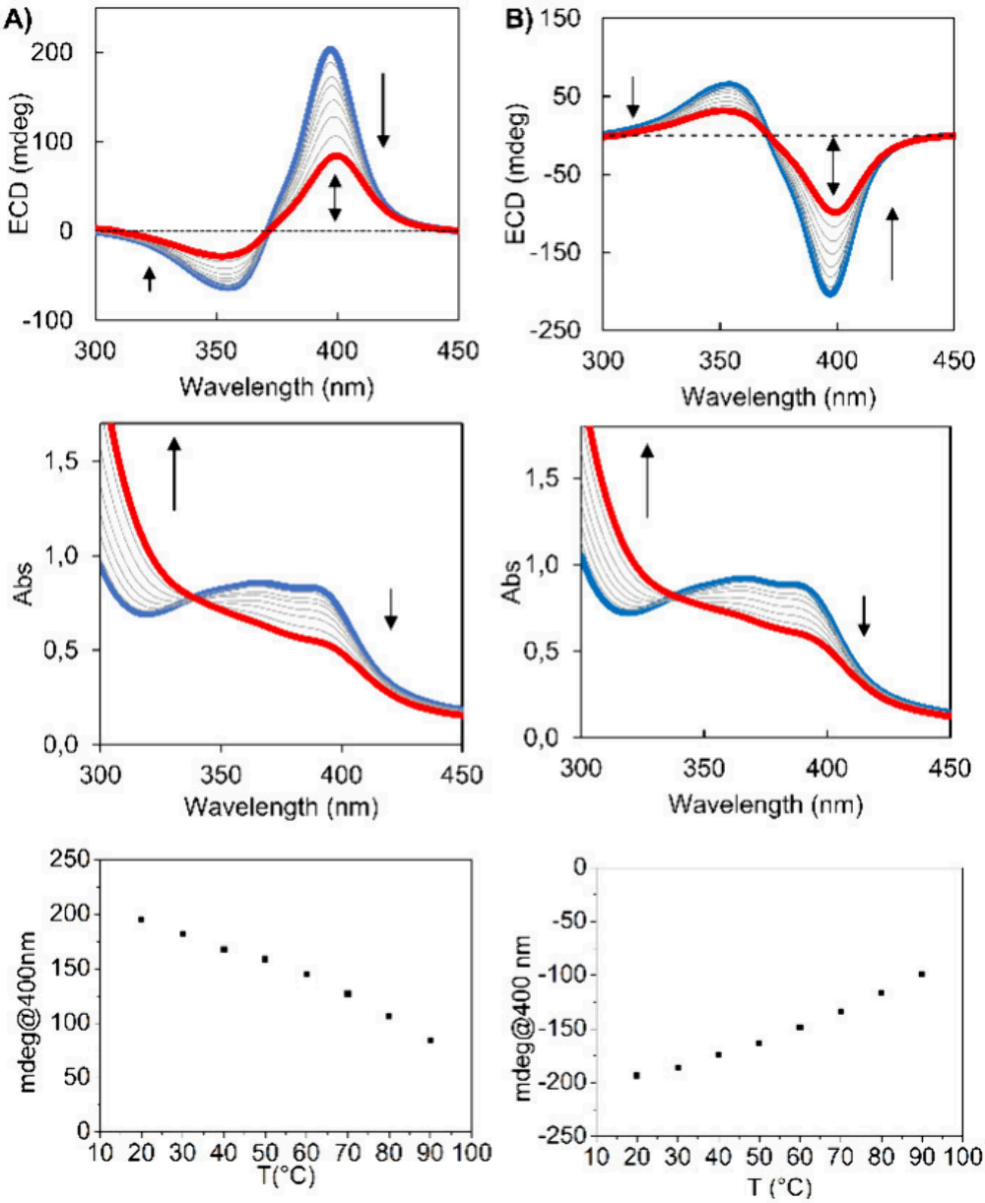
(A) VT-ECD (top) and VT-UV–vis (middle) spectra from 20 °C (blue line) to 90 °C (red line) of **1a**/***D***-**TA**/**Cu** ^**2+**^ ternary assemblies in 15% (v/v) DMSO/H_2_O; bottom: variation of ECD signal at 400 nm as a function of temperature. (B) VT-ECD (top) and VT-UV–vis (middle) spectra from 20 °C (blue line) to 90 °C (red line) of **1a**/***L***-**TA**/**Cu** ^**2+**^ ternary assemblies in 15% (v/v) DMSO/H_2_O; bottom: variation of ECD signal at 400 nm as a function of temperature.

### Computational Studies

To obtain qualitative atomistic insight into the initial mechanisms of aggregate formation, as well as a description of the possible nascent aggregates, we carried out all-atom explicit solvent MD simulations (see Table S1 for the different simulation conditions and Materials and Methods in the SI for the setup). The timespan for the formation of macroscopic supramolecular structures is clearly much longer than that accessible by all-atom MD, so the results described and obtained here refer mainly to the initial recognition among the different components of the final supramolecular assembly. The simulations indicate that the monomers initially start establishing interactions with other monomers with direct contacts among the different triptycene-fused benzimidazoles. The initial aggregates are formed by T-shaped aromatic–aromatic interactions between the bridging benzene rings of two contacting triptycene-benzimidazoles. **TA** molecules play a key role in this mechanism by stabilizing nascent complexes. This is achieved by a direct interaction between negatively charged oxygens of tartaric acid and the positively charged nitrogen atoms on triptycene-benzimidazoles (Figure 11). Interestingly, in the models where **TA** is present, the acid acts as a bridge between two different triptycene-benzimidazole monomers, thus leading to the initial formation of a complex supramolecular architecture (Figure 11). Furthermore, **TA** significantly reduces the number of rotations of the pyridine substituents around the single bond connecting them to the triptycene-fused benzimidazole core (last column of Table S1). A movie of the simulated initial aggregation process is provided as Supporting Information.

**Figure 11:**
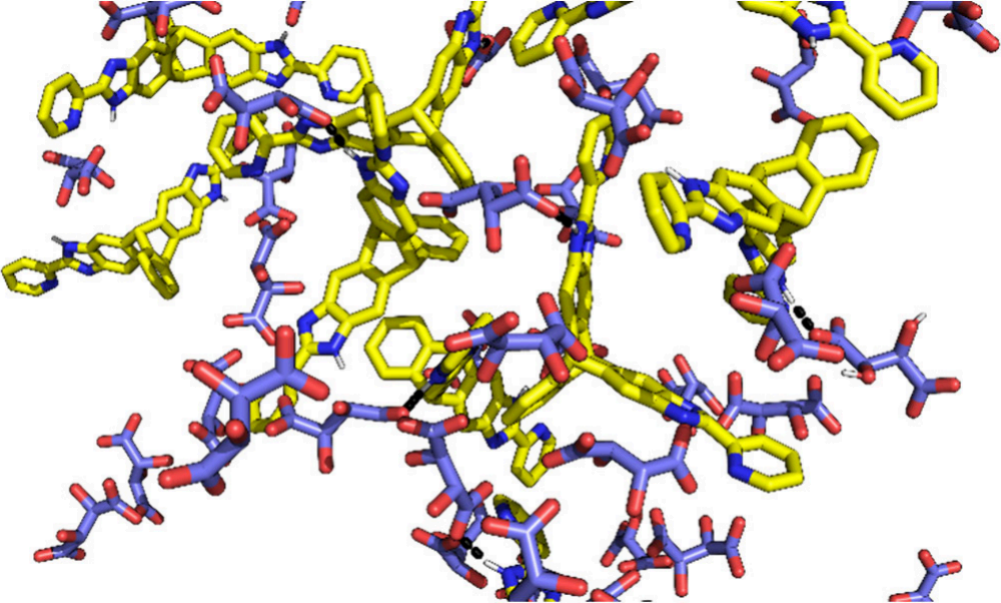
Representative structures of aggregates from MD simulations.

## Conclusions

We reported the synthesis and controlled manipulation of novel chiroptical smart materials composed of “stereochemically fluid” triptycene-fused benzimidazole **1a** and enantiopure **TA** by simply mixing the two components in appropriate solvent/antisolvent mixtures. Besides being a new paradigm for introducing chirality on the triptycene backbone, the simplicity of this method may be used within the high throughput discovery of novel chiroptical materials. The unprecedented use of fused benzimidazole-pyridyl moieties allowed, by the systematic variation of the position of the pyridyl nitrogen atom, one to substantiate the mechanism of chirality transfer. These aggregates combine in one single material a panel of functional properties normally found in different classes of materials: high anisotropy factors (*g*_abs_ up to 1 × 10^–2^), ability to emit circularly polarized light (g_lum_ on the order of 2 × 10^–3^), chiral memory of the stereochemical information imprinted by the chiral auxiliary, self-recovery abilities, and controllable chiroptical activity by external stimuli such as temperature and solvent/antisolvent composition. The aggregates have been characterized by DLS experiments directly as a colloidal dispersion, confirming their nanostructured nature. The aggregates are able to act as chiroptical sensors for metal ions such as Cu^2+^, and the sensing activity can be accomplished in different spectroscopic channels: UV–vis, ECD, fluorescence emission, and CPL. Binary **1a**/**TA** aggregates can be reversibly assembled/disassembled to single molecular components by acting on temperature but not through chemical stimuli such as pH variations or removal of stereochemical information; alternatively, they can be converted to thermally resistant tricomponent assemblies upon the addition of Cu^2+^, thereby modifying the ECD and CPL properties as well.

Each one of these characteristics makes them promising for specific applications: as CPL emitters, chiroptical logic systems, an opportunity to reuse **TA** to trigger continuous chirality circulation and amplification (catalytic supramolecular auxiliary), chiroptical ECD- and CPL-active (bio)­sensors, thermolabile or thermoresistant chiroptical materials, and ideal model systems for investigating supramolecular multicomponent self-assembly processes. Owing to the memory properties, we believe it will be possible to separate the excess of supramolecular auxiliary from chiral aggregates by filtration or precipitation and reutilize it again to induce continuous chirality similar to what happens in catalytic cycles. Upcoming efforts will focus on the isolation of aggregates from the chiral auxiliary and on the high-throughput discovery of new triptycene-fused benzimidazole/chiral acid pairs.

## Supplementary Material





## Notes

Movie of the initial formation of the aggregates **1a**/***L***-**TA** simulated by molecular dynamics (MP4)
Detailed procedures for nanoparticle preparations, methodologies for spectroscopic and VT studies, methods for computational studies, and additional spectroscopic graphs and details (PDF)

## References

[1] Lehn J.-M. (2015). Perspectives
in Chemistry—Aspects of Adaptive Chemistry and Materials. Angew. Chem. Int. Ed..

[2] de
Jong J. J. D., Lucas L. N., Kellogg R. M., van Esch J. H., Feringa B. L. (2004). Reversible Optical Transcription of Supramolecular
Chirality into Molecular Chirality. Science.

[3] Torsi L., Farinola G. M., Marinelli F., Tanese M. C., Omar O. H., Valli L., Babudri F., Palmisano F., Zambonin P. G., Naso F. (2008). A Sensitivity-Enhanced
Field-Effect
Chiral Sensor. Nat. Mater..

[4] Zhao Y., Askarpour A. N., Sun L., Shi J., Li X., Alù A. (2017). Chirality
Detection of Enantiomers Using Twisted Optical
Metamaterials. Nat. Commun..

[5] Shen J., Okamoto Y. (2016). Efficient Separation of Enantiomers Using Stereoregular
Chiral Polymers. Chem. Rev..

[6] Huang S., Yu H., Li Q. (2021). Supramolecular
Chirality Transfer toward Chiral Aggregation:
Asymmetric Hierarchical Self-Assembly. Adv.
Sci..

[7] Wang J., Feringa B. L. (2011). Dynamic
Control of Chiral Space in a Catalytic Asymmetric
Reaction Using a Molecular Motor. Science.

[8] Dorca Y., Greciano E. E., Valera J. S., Gómez R., Sánchez L. (2019). Hierarchy of Asymmetry in Chiral
Supramolecular Polymers:
Toward Functional, Helical Supramolecular Structures. Chem.—Eur. J..

[9] Yan Y., Wang R., Qiu X., Wei Z. (2010). Hexagonal Superlattice
of Chiral Conducting Polymers Self-Assembled by Mimicking β-Sheet
Proteins with Anisotropic Electrical Transport. J. Am. Chem. Soc..

[10] Wang L., Urbas A. M., Li Q. (2020). Nature-Inspired Emerging Chiral Liquid
Crystal Nanostructures: From Molecular Self-Assembly to DNA Mesophase
and Nanocolloids. Adv. Mater..

[11] Yang Y., Zhang Y., Wei Z. (2013). Supramolecular
Helices: Chirality
Transfer from Conjugated Molecules to Structures. Adv. Mater..

[12] Yoo S., Park Q.-H. (2018). Metamaterials
and Chiral Sensing: A Review of Fundamentals
and Applications. Nanophotonics.

[13] Hou J., Toyoda R., Meskers S. C. J., Feringa B. L. (2022). Programming and
Dynamic Control of the Circular Polarization of Luminescence from
an Achiral Fluorescent Dye in a Liquid Crystal Host by Molecular Motors. Angew. Chem. Int. Ed..

[14] Ma L.-L., Hu W., Zheng Z.-G., Wu S.-B., Chen P., Li Q., Lu Y.-Q. (2019). Light-Activated Liquid Crystalline Hierarchical Architecture Toward
Photonics. Adv. Optical Mater..

[15] Xing P., Tham H. P., Li P., Chen H., Xiang H., Zhao Y. (2018). Environment-Adaptive
Coassembly/Self-Sorting and Stimulus-Responsiveness
Transfer Based on Cholesterol Building Blocks. Adv. Sci..

[16] Xing P., Zhao Y. (2018). Controlling Supramolecular
Chirality in Multicomponent Self-Assembled
Systems. Acc. Chem. Res..

[17] Boiani M., Gonzalez M. (2005). Imidazole and Benzimidazole
Derivatives as Chemotherapeutic
Agents. Mini Rev. Med. Chem..

[18] Zhou X., Jin Q., Zhang L., Shen Z., Jiang L., Liu M. (2016). Self-Assembly
of Hierarchical Chiral Nanostructures Based on Metal-Benzimidazole
Interactions: Chiral Nanofibers, Nanotubes, and Microtubular Flowers. Small.

[19] Xing C., Deng J., Fu W., Li J., Xu L., Sun R., Wang D., Li C., Liang K., Gao M., Kong B. (2022). Interfacially Super-Assembled Benzimidazole Derivative-Based Mesoporous
Silica Nanoprobe for Sensitive Copper (II) Detection and Biosensing
in Living Cells. Chem.—Eur. J..

[20] Zhao J., Hao A., Xing P. (2021). Enhancing
Optical Activities of Benzimidazole Derivatives
through Coassembly for High-Efficiency Synthesis of Chiroptical Nanomaterials
and Accurate Ee% Detection of Natural Acids. ACS Appl. Mater. Interfaces.

[21] Zhao J., Hao A., Xing P. (2021). Quantitative Chiral Sensing of Organic Acids by Benzimidazole
Derivatives through H-Bond Coassembly. J. Mater.
Chem. C.

[22] Xing P., Li Y., Xue S., Fiona Phua S. Z., Ding C., Chen H., Zhao Y. (2019). Occurrence
of Chiral Nanostructures Induced by Multiple Hydrogen
Bonds. J. Am. Chem. Soc..

[23] Zhao J., Liu Y., Hao A., Xing P. (2020). High-Throughput Synthesis of Chiroptical Nanostructures from Synergistic
Hydrogen-Bonded Coassemblies. ACS Nano.

[24] Woźny M., Mames A., Ratajczyk T. (2022). Triptycene
Derivatives: From Their
Synthesis to Their Unique Properties. Molecules.

[25] Chong J. H., MacLachlan M. J. (2009). Iptycenes
in Supramolecular and Materials Chemistry. Chem.
Soc. Rev..

[26] Preda G., Nitti A., Pasini D. (2020). Chiral Triptycenes
in Supramolecular
and Materials Chemistry. ChemistryOpen.

[27] Khan Md. N., Wirth T. (2021). Chiral Triptycenes:
Concepts, Progress and Prospects. Chem.—Eur.
J..

[28] Preda G., Mobili R., Ravelli D., Amendola V., Pasini D. (2024). Homoconjugation and Tautomeric Isomerism
in Triptycene-Fused Pyridylbenzimidazoles. J.
Org. Chem..

[29] Feng X., Kawabata K., Kaufman G., Elimelech M., Osuji C. O. (2017). Highly Selective Vertically Aligned
Nanopores in Sustainably
Derived Polymer Membranes by Molecular Templating. ACS Nano.

[30] Xiong J.-F., Luo S.-H., Huo J.-P., Liu J.-Y., Chen S.-X., Wang Z.-Y. (2014). Design, Synthesis, and Characterization of 1,3,5-Tri­(1H-Benzo­[d]­Imidazol-2-Yl)­Benzene-Based
Fluorescent Supramolecular Columnar Liquid Crystals with a Broad Mesomorphic
Range. J. Org. Chem..

[31] Ammenhäuser R., Lupton J. M., Scherf U. (2024). Chain-Length
Dependence of the Optical
Activity of Helical Triptycene-Based π-Conjugated Ladder Polymers. Adv. Optical Mater..

[32] Zhao J.-S., Ruan Y.-B., Zhou R., Jiang Y.-B. (2011). Memory of chirality
in J-type aggregates of an achiral perylene dianhydride dye created
in a chiral asymmetric catalytic synthesis. Chem. Sci..

[33] Witte F., Rietsch P., Sinha S., Krappe A., Joswig J.-O., Gotze J. P., Nirmalananthan-Budau N., Resch-Genger U., Eigler S., Paulus B. (2021). Fluorescence Quenching
in J-Aggregates
through the Formation of Unusual Metastable Dimers. J. Phys. Chem. B.

[34] Nieto-Ortega B., García F., Longhi G., Castiglioni E., Calbo J., Abbate S., Navarrete J. T. L., Ramírez F. J., Ortí E., Sánchez L., Casado J. (2015). On the Handedness of Helical Aggregates
of *C*
_3_ Tricarboxamides: A Multichiroptical
Characterization. Chem. Commun..

[35] González-Sánchez M., Mayoral M. J., Vázquez-González V., Paloncýová M., Sancho-Casado I., Aparicio F., de Juan A., Longhi G., Norman P., Linares M., González-Rodríguez D. (2023). Stacked or
Folded? Impact of Chelate Cooperativity on the Self-Assembly Pathway
to Helical Nanotubes from Dinucleobase Monomers. J. Am. Chem. Soc..

[36] Smulders M. M. J., Nieuwenhuizen M. M. L., de Greef T. F. A., van
der Schoot P., Schenning A. P. H. J., Meijer E. W. (2010). How to Distinguish
Isodesmic from Cooperative Supramolecular Polymerisation. Chem.—Eur. J..

[37] Greenfield N. J. (2006). Using Circular
Dichroism Collected as a Function of Temperature to Determine the
Thermodynamics of Protein Unfolding and Binding Interactions. Nat. Protoc..

[38] Huynh K., Partch C. L. (2015). Analysis of Protein Stability and Ligand Interactions
by Thermal Shift Assay. Current Protocols in
Protein Science.

[39] Miles A. J., Wallace B. A. (2016). Circular Dichroism Spectroscopy of
Membrane Proteins. Chem. Soc. Rev..

[40] Miao T., Cheng X., Zhang G., Wang Y., He Z., Wang Z., Zhang W. (2023). Self-Recovery
of Chiral Microphase
Separation in an Achiral Diblock Copolymer System. Chem. Sci..

[41] Ohira A., Okoshi K., Fujiki M., Kunitake M., Naito M., Hagihara T. (2004). Versatile Helical Polymer Films: Chiroptical Inversion
Switching and Memory with Re-Writable (RW) and Write-Once Read-Many
(WORM) Modes. Adv. Mater..

[42] Miao T., Cheng X., Ma H., He Z., Zhang Z., Zhou N., Zhang W., Zhu X. (2021). Transfer,
Amplification,
Storage, and Complete Self-Recovery of Supramolecular Chirality in
an Achiral Polymer System. Angew. Chem., Int.
Ed..

[43] Chen J., Xu L., Lin X., Chen R., Yu D., Hong W., Zheng Z., Chen X. (2018). Self-Healing Responsive Chiral Photonic
Films for Sensing and Encoding. J. Mater. Chem.
C.

[44] Preda G., Mobili R., La Cognata S., Toma L., Pasini D., Amendola V. (2022). Chiroptical sensing
of perrhenate in aqueous media
by a chiral organic cage”. Chem. Commun..

[45] Agnes M., Nitti A., Vander Griend D. A., Dondi D., Merli D., Pasini D. (2016). A chiroptical molecular
sensor for ferrocene. Chem. Commun..

[46] Caricato M., Leza N. J., Roy K., Dondi D., Gattuso G., Shimizu L. S., Vander
Griend D. A., Pasini D. (2013). A Chiroptical Probe
for Sensing Metal Ions in Water. Eur. J. Org.
Chem..

[47] Preda G., La Cognata S., Pedraza-González L., Carlier L., Kolb M., Pescitelli G., Amendola V., Armspach D., Pasini D. (2025). Manipulating stereo-communication in binaphthol-bridged
α- and β-cyclodextrins to develop β-selective chiroptical
pH switching and anion sensing in water. Org.
Chem. Front..

[48] Jhun B. H., Park S. Y., You Y. (2023). Molecular
Sensors Producing Circularly
Polarized Luminescence Responses. Dyes Pigments.

[49] Reine P., Ortuno A. M., Resa S., Alvarez de Cienfuegos L., Blanco V., Ruedas-Rama M. J., Mazzeo G., Abbate S., Lucotti A., Tommasini M., Guisan-Ceinos S., Ribagorda M., Campana A. G., Mota A., Longhi G., Miguel D., Cuerva J. M. (2018). OFF/ON Switching
of Circularly Polarized
Luminescence by Oxophilic Interaction of Homochiral Sulfoxide-Containing
o-OPEs with Metal Cations. Chem. Commun..

