# Supplementary material for: Chirality Transfer, Memory and Sensing Activated by a Supramolecular Chiral Auxiliary Approach in Nanostructured, Tautomerically Prochiral Triptycene-Fused Benzimidazoles
Source: J Am Chem Soc. 2026 May 7;148(23):23923–31. doi: 10.1021/jacs.6c03689 (PMC13281537; doi:10.1021/jacs.6c03689)
Supplement: Supplementary file 2 [file ja6c03689_si_002.pdf]

Supporting information for:

Chirality Transfer, Memory and Sensing Activated by a Supramolecular Chiral Auxiliary Approach in Nanostructured, Tautomerically Prochiral Triptycene-Fused Benzimidazoles

Giovanni Preda,[1,‡] Riccardo Mobili,[1,‡] Giovanna Longhi,[2] Massimiliano Meli,[3] Giorgio Colombo,[1,3] Valeria Amendola[1,*] and Dario Pasini[1,*]

1) Department of Chemistry and INSTM Research Unit, University of Pavia Via Taramelli 12, 27100 Italy,

2) Dipartimento di Medicina Molecolare e Traslazionale, University of Brescia, Viale Europa 11, 25123 Brescia (Italy) and INO-CNR, Research Unit Brescia, c/o CSMT via Branze, 45, 25123 Brescia (Italy)

3) Istituto di Scienze e Tecnologie Chimiche "Giulio Natta" – “SCITEC” CNR, Via Mario Bianco 9, 20131 Milano (Italy)

1. Materials and Methods S2

2. Additional Spectroscopy S4

3. Additional References S18

1. Materials and Methods

Synthesis. All reagents and solvents were purchased from Merck, Fluorochem, Alfaesar, TCI or Carlo Erba. Syntheses of compounds **1a**, **1b**, **1c**, **2** and **3** have been previously reported.[S1,S2]

General procedure for the supramolecular synthesis of binary chiral aggregates. Here we report a typical standard procedure for obtaining binary chiral aggregates **1a**/***L*-TA** 1/125 in 15% (v/v) DMSO/H_2O solvent mixture. Two concentrated stock solutions of **1a** and ***L*-TA** in DMSO, 0.0352 M and 0.696 M respectively, are prepared and used for making several batches of aggregates. Using micropipettes, 6.0 μL (2.11·x 10^{-7} mol) and 37.8 μL (2.64·x 10^{-5} mol, 125 equiv.) of stock solutions of **1a** and ***L*-TA** were taken out and placed in a vial. After that, 556 μL of DMSO and 3400 μL of distilled H_2O were added to the vial to obtain a 15% (v/v) DMSO/H_2O solution. Finally, after gently shaking and capping, the vial was stored in the refrigerator at 4°C for at least one week. At this point the mixture appears as a transparent colloidal suspension and is used for all further characterization/manipulations.

General procedure for the supramolecular synthesis of ternary chiral aggregates. Here we report a typical standard procedure for obtaining ternary chiral aggregates **1a**/***L*-TA**/**Cu^{2+}**1/125 in 15% (v/v) DMSO/H_2O solvent mixture. To a vial containing 4 mL of a suspension of chiral **1a**/***L*-TA** aggregates previously formed according to the above-described procedure, 1.61 μL (4.21x 10^{-7} mol, 2 equiv.) of a 0.261 M stock solution of $CuSO_4$ in H_2O is added. The mixture is gently shaken and allowed to rest for 2 days.

Concentrated stock solutions of aqueous metal salts $Ni(SO_4)\cdot 7H_2O$, $CuSO_4$, $CoCl_2\cdot 6H_2O$, $ZnCl_2$, $BaCl_2\cdot 2H_2O$, $Eu(CF_3SO_3)_3$, $CaCl_2$, $Mn(NO_3)_2$ were used as the source of metal ions Ni^{2+}, Cu^{2+}, Co^{2+}, Zn^{2+}, Ba^{2+}, Eu^{3+}, Ca^{2+}, Mn^{2+}, respectively.

One pot procedure for the supramolecular synthesis of ternary chiral aggregates. Here we report a typical ***failed*** one pot procedure for obtaining ternary chiral aggregates **1a**/***L*-TA**/**Cu^{2+}**1/125 in 15% (v/v) DMSO/H_2O solvent mixture. Three concentrated stock solutions of **1a** (0.0352 M), ***L*-TA** (0.696 M) in DMSO and $CuSO_4$ (0.261 M) in H_2O, are prepared. Using micropipettes, 6.0 μL (2.11 x 10^{-7} mol), 37.8 μL (2.64 x 10^{-5} mol, 125 equiv.) and 80.5 μL (2.11 x 10^{-5} mol, 100 equiv.) of stock solution of **1a**, ***L*-TA** and $CuSO_4$ were taken out and placed in a vial. After that, 556 μL of DMSO and 3320 μL of distilled H_2O were added to the vial to obtain a 15% (v/v) DMSO/H_2O solvent mixture. Finally, after gently shaking and capping, the vial was stored in a refrigerator at 4°C for at least one week. At this point the mixture appears as a transparent colloidal suspension.

Variable pH experiments. Increasing amounts (up to *ca* 400 equiv.) of basic (NaOH 0.75 M in H_2O) or acidic (TFA 1 M in H_2O) solutions were added to 20 mL of suspension of aggregates **1a**/**L-TA** previously

assembled according to the above method until the final pHs of 1.7 and 11.5, respectively, were reached.

UV-vis, ECD and fluorescence emission spectra. UV−vis spectra were collected using an Agilent Varian Cary 50 SCAN spectrophotometer, with quartz cuvette of the 1 cm path length at 25°C. Emission spectra were collected using an Agilent Cary Eclipse Fluorescence Spectrophotometer, with a quartz cuvette of 1 cm path length at 25°C. ECD spectra were collected on a JASCO J1500 spectropolarimeter equipped with a Peltier temperature controller. A quartz cell of 1 cm optical length was used for all measurements. All spectra were recorded using a scanning speed of 200 nm min^{-1}, a step size of 1 nm, a bandwidth of 2 nm, a response time of 2 s, and an accumulation of 2 scans. The spectra were background-corrected using spectra of respective solvents recorded under the same conditions. VT-ECD/UV-vis measurements were carried out by using a Peltier-temperature programmer using measurement parameters listed above. For VT-ECD experiments in the presence of excess Cu^{2+}, stirring at 600 rpm was applied by magnetic stirrer programmer to prevent flocculation within the samples. Denaturation melting temperatures (T_m) have been derived by non-linear fitting of ellipticity vs temperature experimental data with a Boltzmann sigmoidal function.[S3,S4] The anisotropy absorption factor g_{abs} was calculated using the following formula:

g_{abs}= CD / (Abs x 32980)

Dynamic Light Scattering measurements (DLS). Dimension of the aggregates in 15% (v/v) DMSO/H_2O solvent mixture were performed with a Zetasizer Nano-ZS90 (source: polarized He-Ne laser, 30 mW output power, vertically polarized) on 1 mL of colloidal suspension in a 1 cm quartz cuvette measuring at an angle of 13° and at a temperature of 25 °C. VT-DLS experiments were performed with a Peltier temperature controller integrated in the instrument. Viscosity and refractive index of the solvent mixture were corrected for the DMSO content using literature data.[S5]

Circularly polarized luminescence spectra. CPL spectra were collected on the home-built apparatus[S6] in the range of 400-650 nm, with 100 nm/min scanning speed, with a 90° geometry, an excitation wavelength of 350 nm (brought to the sample with an optical fiber from a Jasco FP8000 fluorimeter), and 30 scans accumulation. The glum ratio was evaluated through:

$$g_{lum} = 2\frac{I_L - I_R}{I_L + I_R},$$

I_L and I_R being the “intensities” of emitted left and right circularly polarized light provided by our instrument.

Computational Studies. Triptycene-fused benzimidazole monomers **1a** and L-tartaric acid (**L-TA**) were parameterized with the all-atom CGenFF force field using the CHARMM-GUI suite[S6—S10] Parameters for dimethyl sulfoxide (DMSO) and counterions were already available in the Charmm36 force field. A

summary of the systems simulated, with simulation conditions and the number of molecules used is given in Table S1. The systems were initially assembled by randomly placing **1a** and *L*-tartaric acid (**L-TA**) monomers in simulation boxes, using the Multicomponent Assembler utility in the CHARMM-GUI web interface.[S11] DMSO was also added to reflect experimental conditions. The boxes were then filled with TIP3P water molecules.[S12]

Next, input files for the all-atom Molecular Dynamics (MD) simulation were created and formatted for use in the AMBER 22 suite of programs. All MD simulations and standard structural analyses were performed with the AMBER 22 suite of programs,[S13] and the CUDA implementation for GPUs.

The positions of the water molecules and the counterions were optimized by a restrained minimization consisting of 2500 steps of steepest descent followed by 2500 steps of conjugate gradient minimization. The minimized systems were then equilibrated at 300 K for 10 ns using Langevin coupling with gamma equal to 1 ps^{-1}. After this step, the relaxed systems were simulated in the NPT ensemble at 1 atm using the Montecarlo barostat with Langevin dynamics coupling with gamma equal to 1 ps^{-1}.[S14] The full particle mesh Ewald method was used for electrostatics.[S15] The SHAKE algorithm was used to constrain all covalent bonds involving hydrogen atoms.[S16] A 2-fs time step and a 12 Å cutoff were used to constrain non-bonded van der Waals interactions. The extent of the various production runs is reported in Table S1.

Table S1. Simulation conditions summary of structural parameters from MD. Column reports the simulation label. Columns from 2 to 7 report the number of molecule of: **1a**, **L-TA**, H_2O, DMSO and the counter ions K^+ and Cl^- used to keep the electroneutrality of the system at 0.015M ions strength. Column 8 reports the simulation time in nanoseconds. Column 9 defines the dimensions of the simulation box. Column 10 reports the average number of molecules in anti conformation (see also Figure 10). Column 11 reports the average rotation time of benzimidazole-pyridine axis done in the unit time during the simulation.

Simulation Label	Num **1a**	Num **TA**	Num **H_2O**	Num **DMSO**	Num K^+	Num CL^-	Sim Time (ns)	Vol (nm^3)	Molecules in Anti Conformation	Rotation Time (ns)
**1**	10	0	16006	0	45	65	358	483.5	5	0.0478
**2**	10	0	26111	900	62	82	237	896.8	5	0.0540
**3**	10	400	32185	300	849	89	202	1050.1	7.2	0.00338
**4**	15	400	24218	300	805	65	490	813.1	8	0.00371
**5**	20	400	24866	300	787	67	710	835.7	9.7	0.00358

2. Additional Spectroscopy

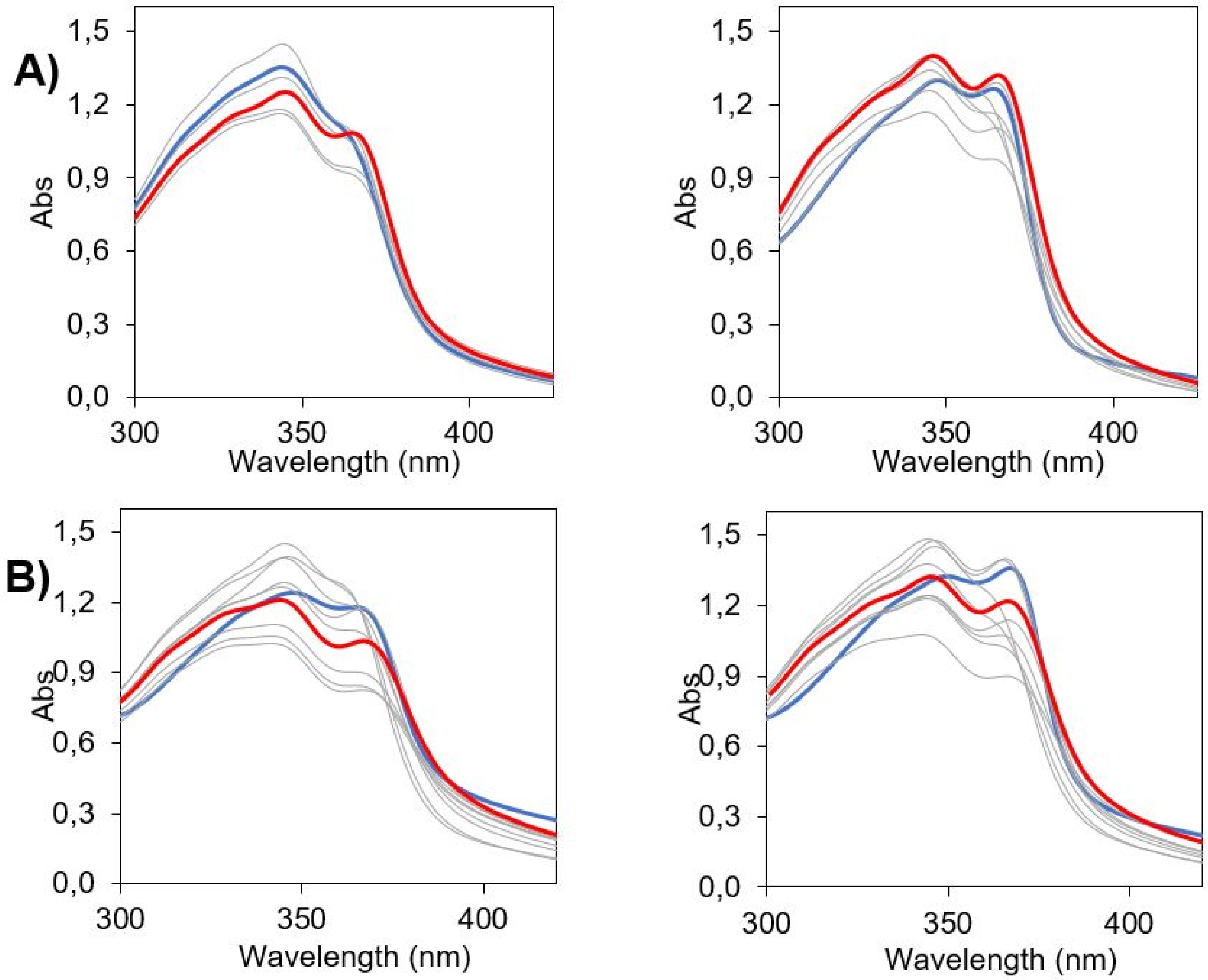

Figure S1. A) UV-vis spectra of **1a**/***D*-TA** (left) and **1a**/***L*-TA** (right) aggregates with ratios from 1/1 (blue line) to 1/150 equiv. (red line) of **TA** in 15% (v/v) DMSO/H_2O. **B) 1a**/***D*-TA** (left) and **1a**/***L*-TA** (right) aggregates with ratios from 1/1 (blue line) to 1/150 equiv. (red line) of **TA** in 11% (v/v) THF/H_2O.

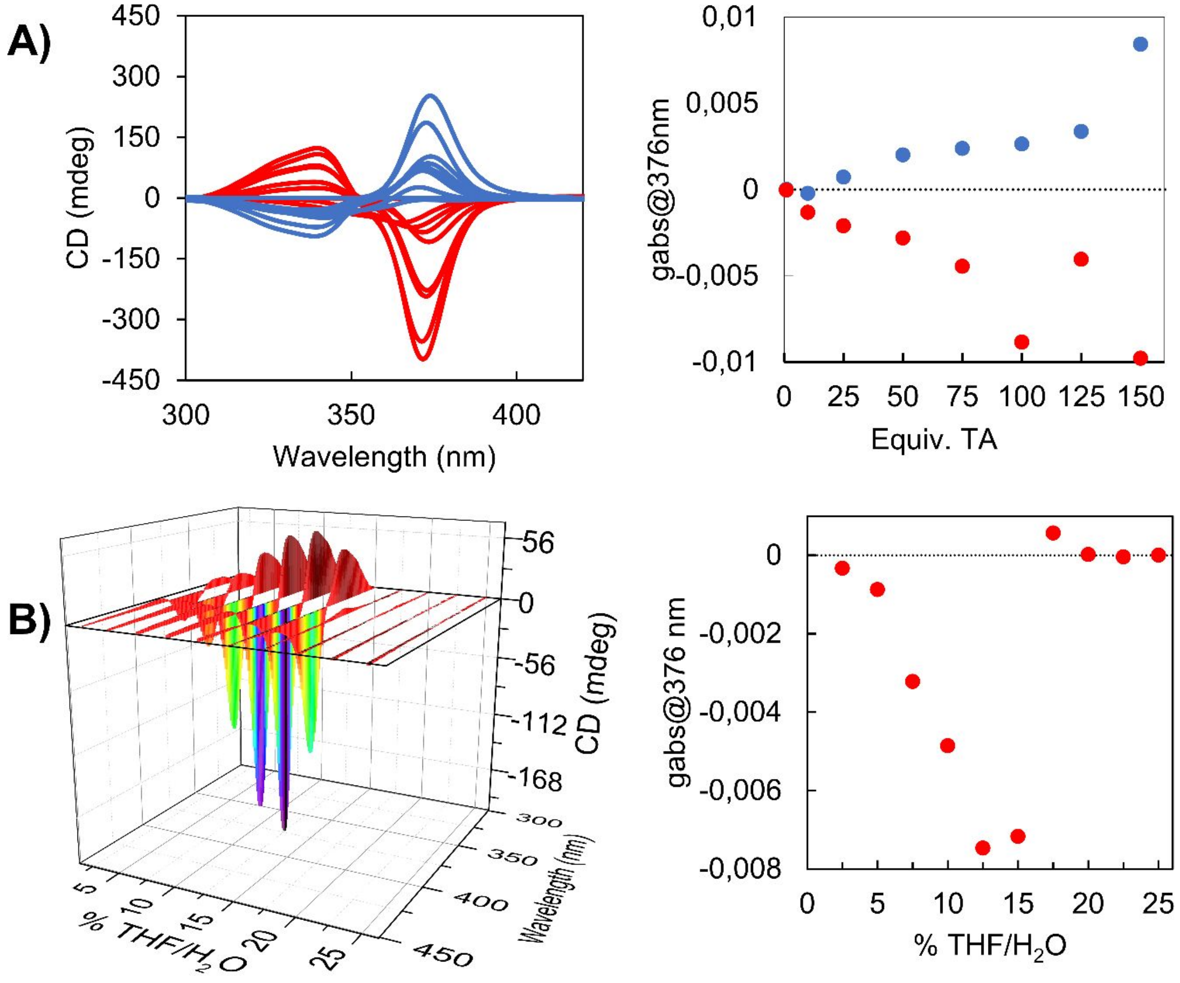

Figure S2. A) Left: ECD spectra (left) of **1a/*D*-TA** (blue) and **1a/*L*-TA** (red) aggregates with ratios from 1/1 to 1/150 equiv. of **TA** in 11% (v/v) THF/H_2O solvent mixture and (right) trend of g_{abs} at 376 nm versus equiv. of **TA**. **B)** Left: 3D and 2D plots of ECD spectra of **1a/*L*-TA** 1/75 aggregates in different (v/v) THF/H_2O solvent mixtures and (right) trend for g_{abs} (376 nm) as a function of (v/v) THF/H_2O solvent mixtures.

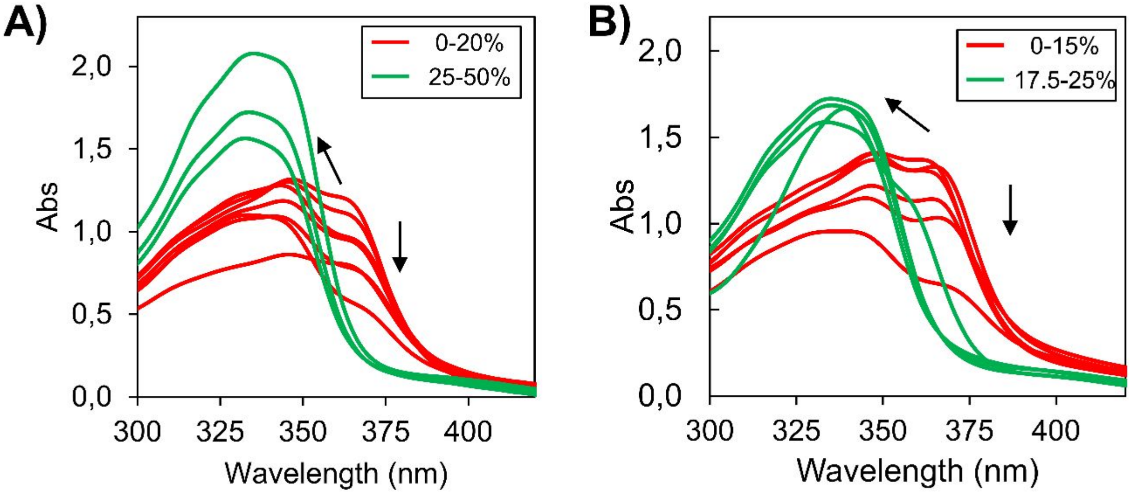

Figure S3. A) UV-vis spectra of **1a/*L*-TA** 1/75 aggregates in different (v/v) DMSO/H_2O solvent mixtures. **B)** UV-vis spectra of **1a/*L*-TA** 1/75 aggregates in different (v/v) THF/H_2O solvent mixtures.

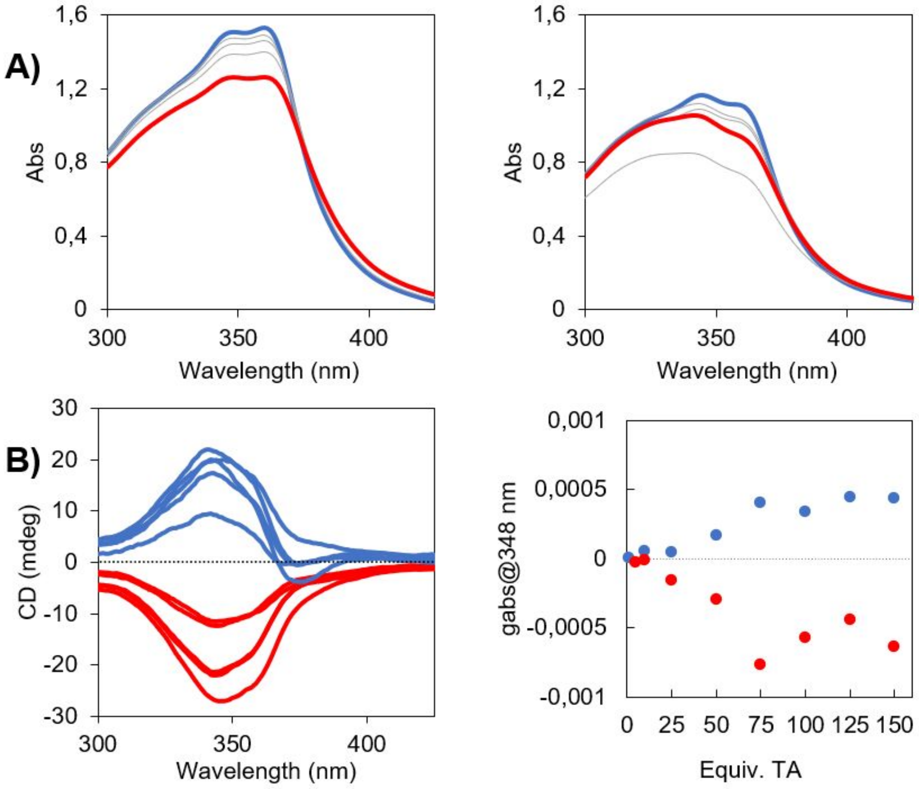

Figure S4. A) UV-vis (bottom) spectra of **1b**/***D*-TA** (blue) and **1b**/***L*-TA** (red) aggregates with ratios from 1/1 (blue lines) to 1/150 equiv. (red lines) of **TA** in 15% (v/v) DMSO/H_2O. **B)** Left: ECD spectra of **1b**/***D*-TA** (blue) and **1b/*L*-TA** (red) aggregates with ratios from 1/1 to 1/150equiv. of **TA** in 15% (v/v) DMSO/H_2O and (right) trend of g_{abs} at 348 nm versus equiv. of **TA**.

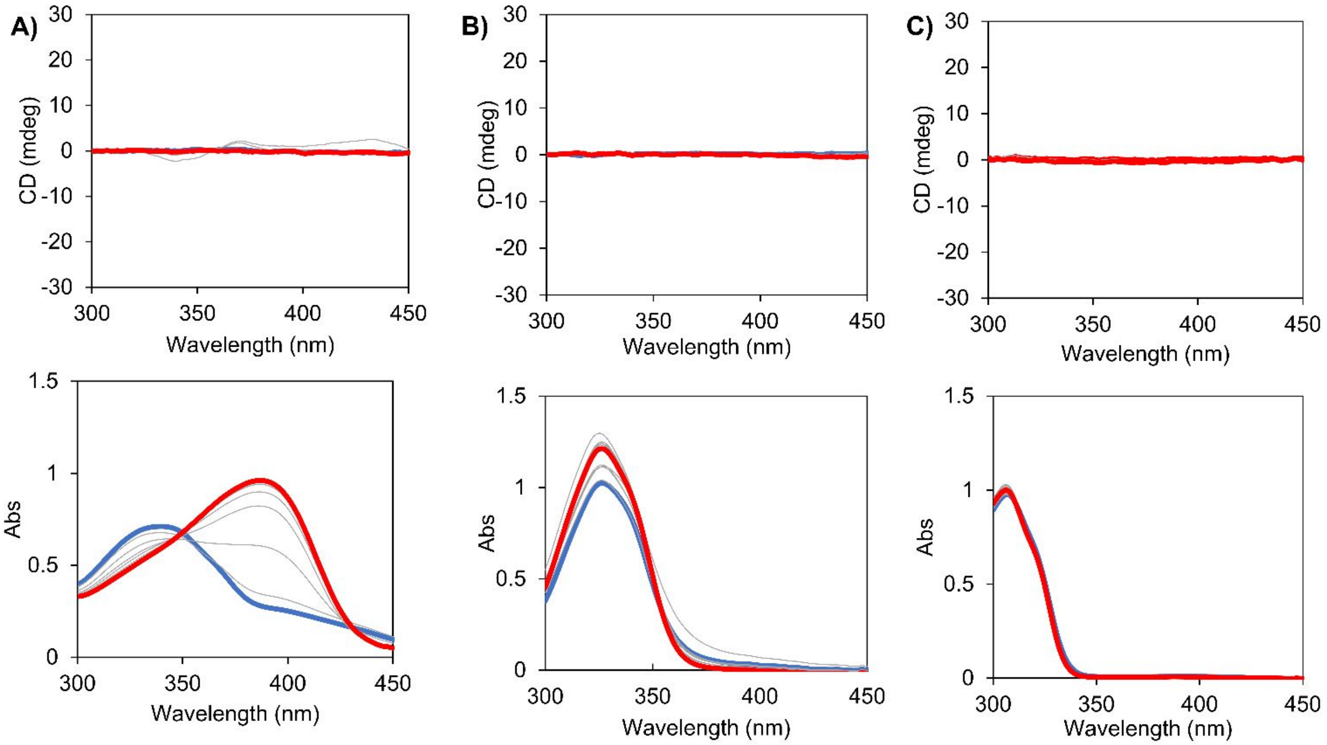

Figure S5. **A)** ECD (top) and UV-vis (bottom) spectra of **1c**/***L*-TA** mixtures in 15% (v/v) DMSO/H_2O, for molar ratios **1c**/***L*-TA** from 1/2.5 (blue line) to 1/150 (red line), **[1c]** = 5 x·10^{-5} M. **B)** ECD (top) and UV-vis (bottom) spectra of **2**/***L*-TA** mixtures in 15% (v/v) DMSO/H_2O, for molar ratios **2**/***L*-TA** from 1/2.5 (blue line) to 1/225 (red line); **[2]** = 7 x·10^{-5} M; **C)** ECD (top) and UV-vis (bottom) spectra of **3**/***L*-TA** mixtures in 15% (v/v) DMSO/H_2O, for molar ratios **3**/***L*-TA** from 1/2.5 (blue line) to 1/225 (red line); **[3]** = 8 x·10^{-5} M.

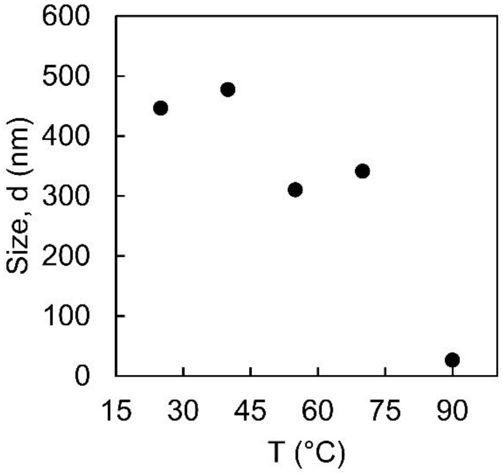

Figure S6. trend of aggregate sizes as a function of temperature (from 25 to 90°C), as obtained from DLS.

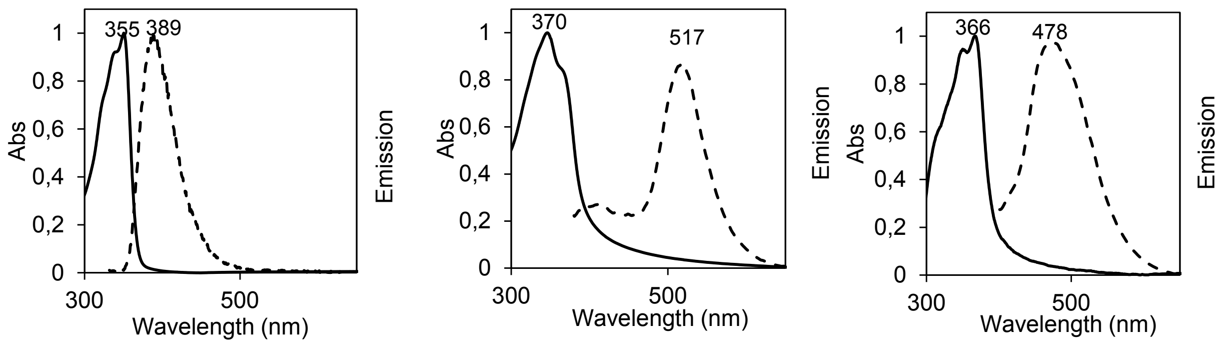

Figure S7. Left Normalized UV-vis and fluorescence emission spectra of **1a** in DMSO. **Middle** Normalized UV-vis and fluorescence emission spectra of random aggregates composed by **1a** in 15% (v/v) DMSO/H_2O; **Right** Normalized UV-vis and fluorescence emission spectra of chiral aggregates **1a/L-TA** 1/75 in 15% (v/v) DMSO/H_2O mixture.

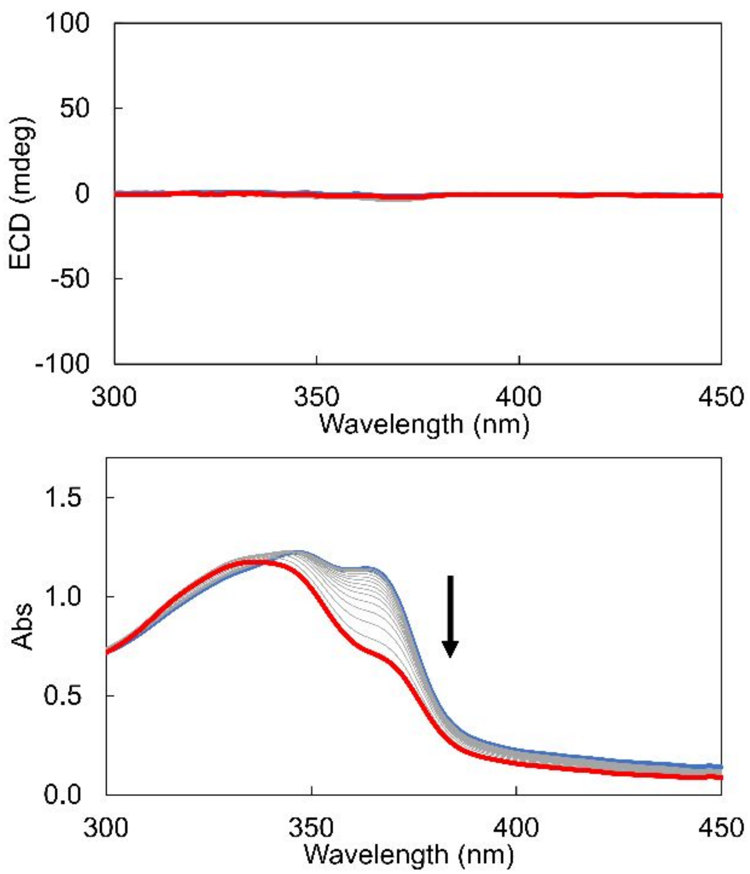

Figure S8. Variable temperature ECD (top) and UV-vis (bottom) spectra of **1a** in 15% (v/v) DMSO/H_2O from 20 °C (blue line) to 90 °C (red line).

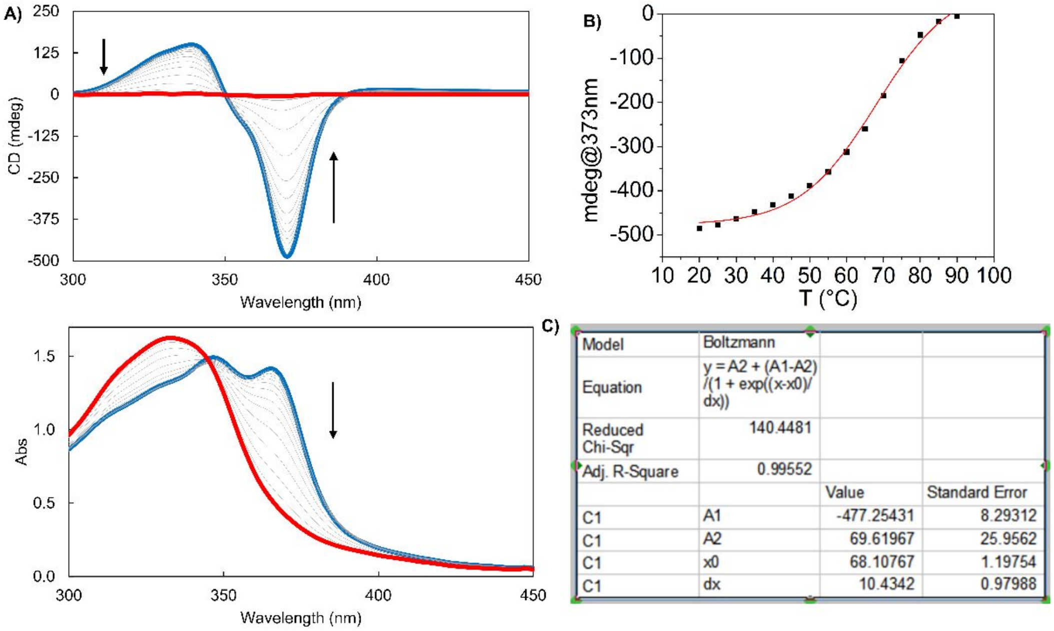

Model	Boltzmann		
Equation	y = A2 + (A1-A2) /(1 + exp((x-x0)/ dx))		
Reduced Chi-Sqr	140.4481		
Adj. R-Square	0.99552		
		Value	Standard Error
C1	A1	-477.25431	8.29312
C1	A2	69.61967	25.9562
C1	x0	68.10767	1.19754
C1	dx	10.4342	0.97988

Figure S9. First round of VT-ECD and VT-Abs spectra from 20°C (blue line) to 90°C (red line) of aggregates **1a**/***L*-TA** 1/125 in 15% (v/v) DMSO/H_2O. **B)** ECD signal at 373 nm as function of temperature; black points = experimental points; red line = Boltzmann fit. **C)** Numerical parameters regarding Boltzmann sigmoidal fit; $T_{melting}$= 68±1 °C.

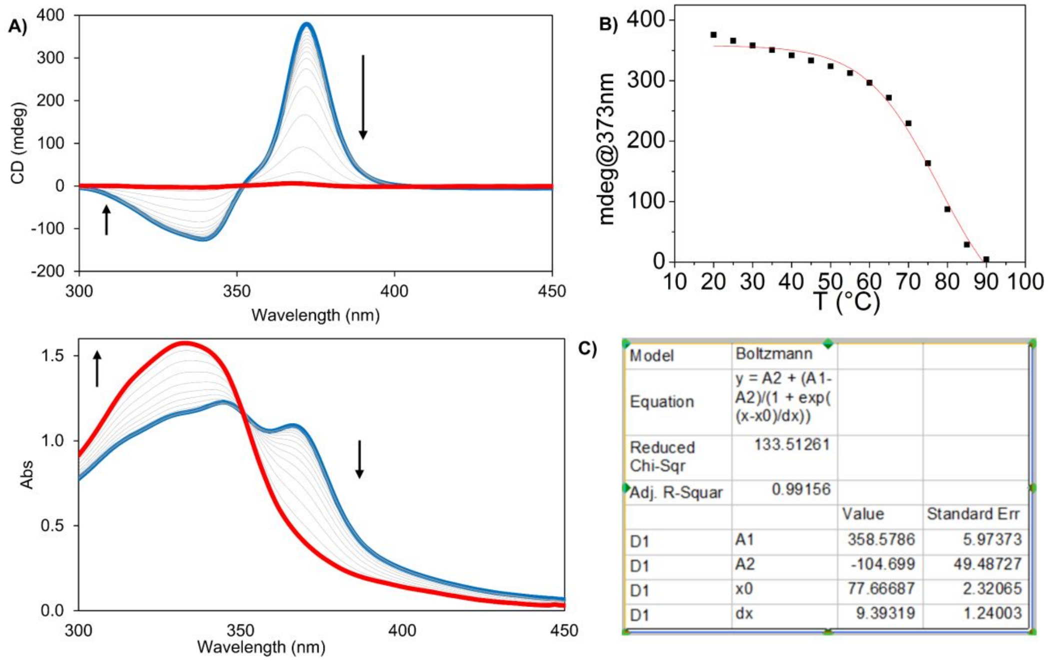

Model	Boltzmann		
Equation	y = A2 + (A1-A2)/(1 + exp((x-x0)/dx))		
Reduced Chi-Sqr	133.51261		
Adj. R-Squar	0.99156		
		Value	Standard Err
D1	A1	358.5786	5.97373
D1	A2	-104.699	49.48727
D1	x0	77.66687	2.32065
D1	dx	9.39319	1.24003

Figure S10. First round of VT-ECD and VT-Abs spectra from 20°C (blue line) to 90°C (red line) of aggregates **1a**/***D*-TA** 1/125 in 15% (v/v) DMSO/H_2O. **B)** ECD signal at 373 nm as function of temperature; black points = experimental points; red line = Boltzmann fit. **C)** Numerical parameters regarding Boltzmann sigmoidal fit; $T_{melting}$= 77±2 °C.

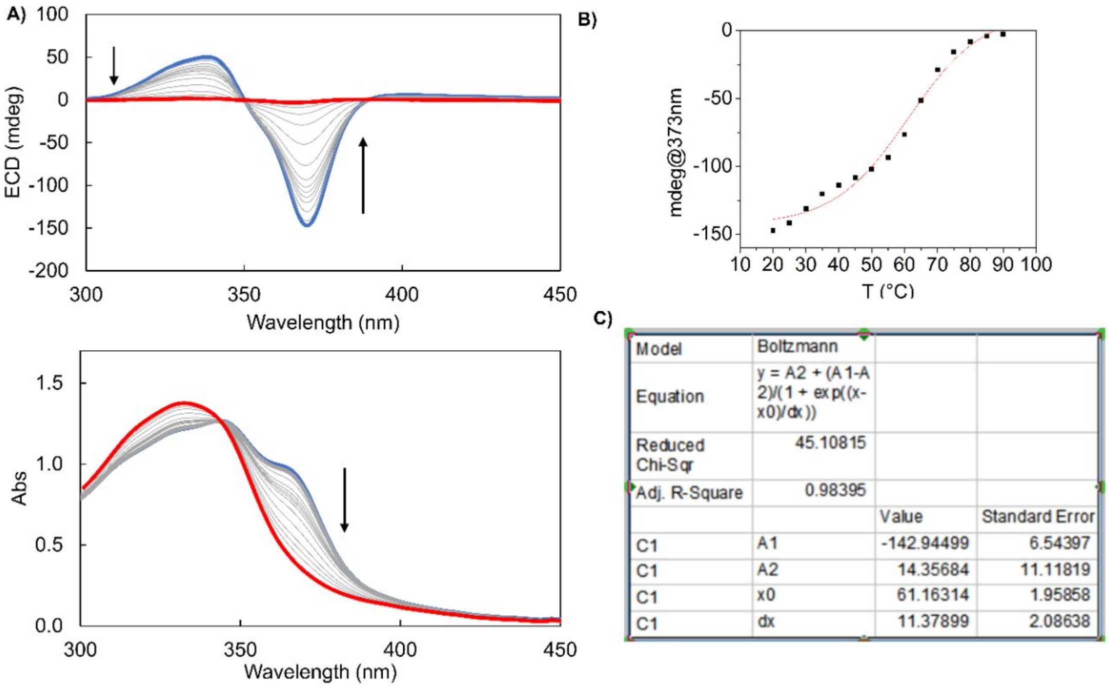

Model	Boltzmann		
Equation	y = A2 + (A1-A2)/(1 + exp((x-x0)/dx))		
Reduced Chi-Sqr	45.10815		
Adj. R-Square	0.98395		
		Value	Standard Error
C1	A1	-142.94499	6.54397
C1	A2	14.35684	11.11819
C1	x0	61.16314	1.95858
C1	dx	11.37899	2.08638

Figure S11. **A)** Second round of VT-ECD and VT-Abs spectra from 20°C (blue line) to 90°C (red line) of aggregates **1a**/***L*-TA** 1/125 in 15% (v/v) DMSO/H_2O. Blue line = spectrum recorded at 20°C, red line = spectrum recorded at 90°C; **B)** ECD signal at 373 nm as function of temperature; black points = experimental points; red line = Boltzmann fit. **C)** Numerical parameters regarding Boltzmann sigmoidal fit; $T_{melting}$= 61±2 °C

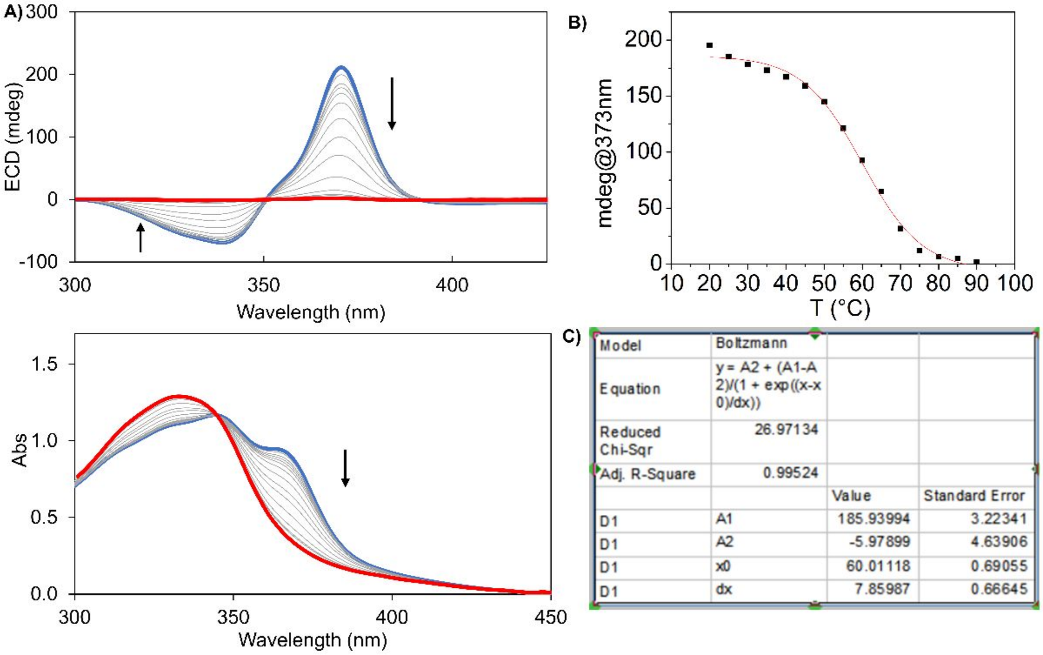

Model	Boltzmann		
Equation	y = A2 + (A1-A2)/(1 + exp((x-x0)/dx))		
Reduced Chi-Sqr	26.97134		
Adj. R-Square	0.99524		
		Value	Standard Error
D1	A1	185.93994	3.22341
D1	A2	-5.97899	4.63906
D1	x0	60.01118	0.69055
D1	dx	7.85987	0.66645

Figure S12. A) Second round of VT-ECD and VT-Abs spectra from 20°C (blue line) to 90°C (red line) of aggregates **1a**/***D*-TA** 1/125 in 15% (v/v) DMSO/H_2O. **B)** ECD signal at 373 nm as function of temperature; black points = experimental points; red line = Boltzmann fit; **C)** Numerical parameters regarding Boltzmann sigmoidal fit; $T_{melting}$= 60±1 °C.

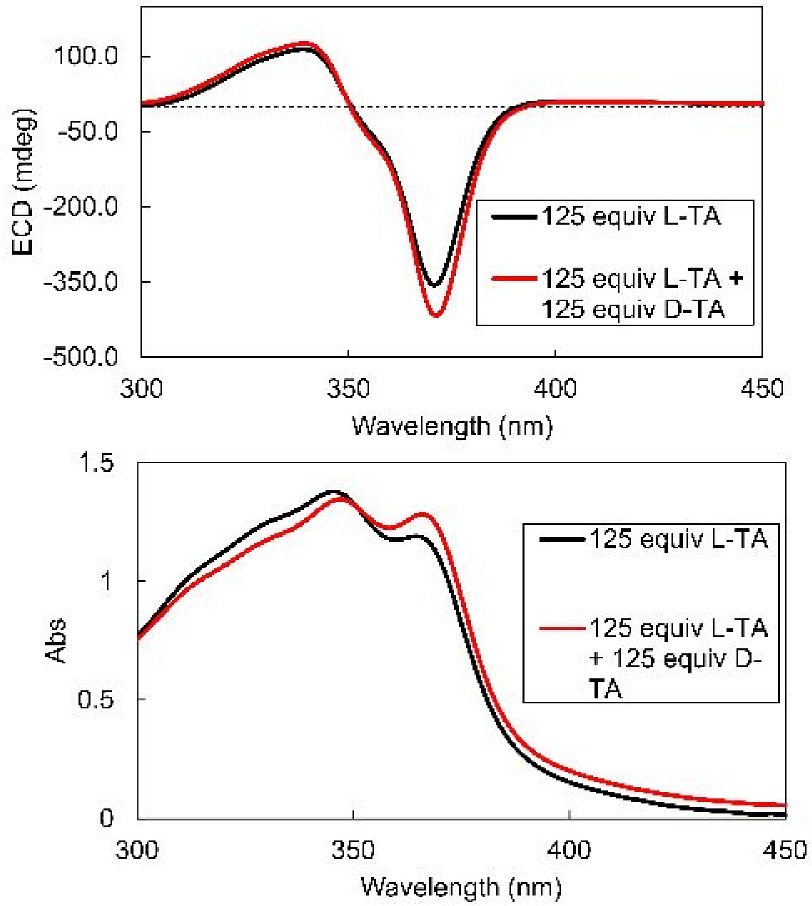

Figure S13. Non-normalized ECD (top) and UV-vis (bottom) spectra of **1a**/***L*-TA** 1/125 aggregates in 15% (v/v) DMSO/H_2O before (black line) and after (red line) addition of additional 125 equiv. of ***D*-TA**. Red spectra have been recorded after 1 month from addition of 125 equiv. of ***D*-TA**.

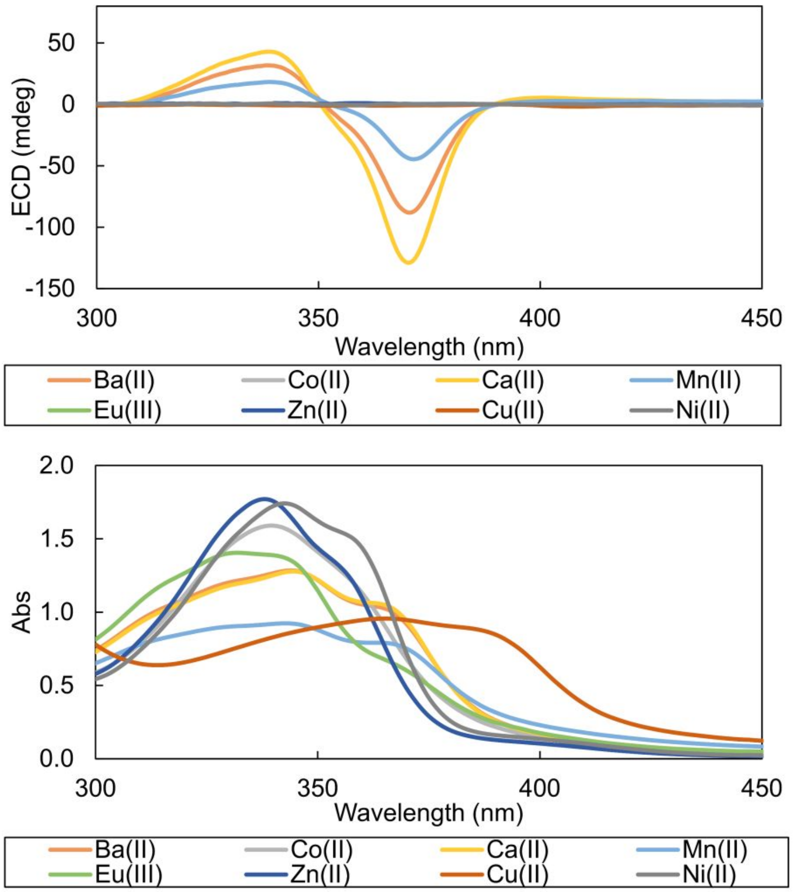

Figure S14. Non-normalized ECD (top) and Uv-vis (bottom) spectra of preliminary assembly experiments for ternary aggregates **1a**/***L*-TA**/**M^{2+}** 1/125/100 in 15% (v/v) DMSO/H_2O. The three components, **1a**, ***L*-TA** and **M^{2+}** were added simultaneously, at 5.2 × 10^{-5}, 6.5 × 10^{-4} and 5.2 × 10^{-4} M concentrations, respectively; spectra recorded after 1 week of aging.

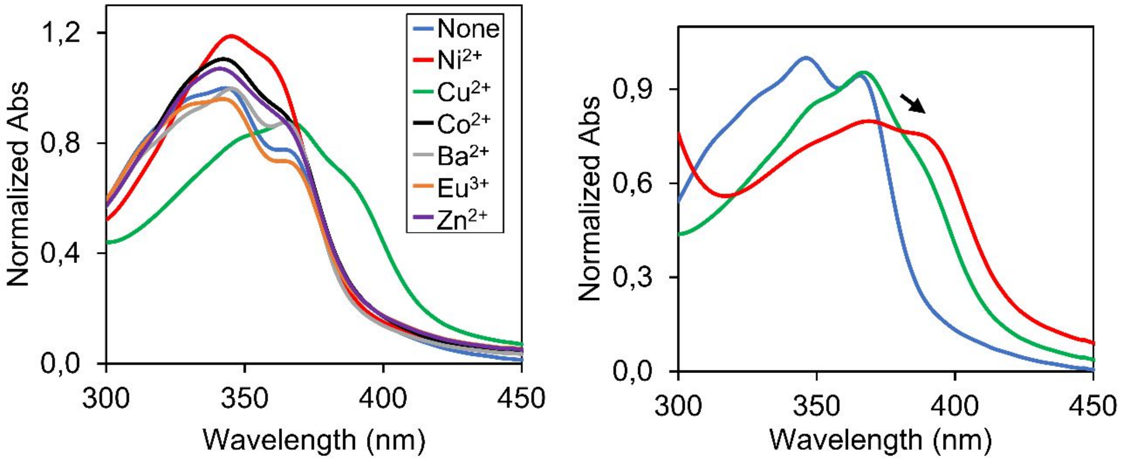

Figure S15 Left Uv-vis spectra of **1a**/***L*-TA** 1/125 aggregates in 15% (v/v) DMSO/H_2O in the presence of 2 equiv. of different metal ions; **Right**: absorption spectra of **1a**/***L*-TA** 1/125 aggregates in 15% (v/v)

DMSO/H_2O in presence of different amounts of Cu^{2+}; blue lines: no Cu^{2+}, green lines: 2 equiv. of Cu^{2+}, red lines: 100 equiv. of Cu^{2+}.

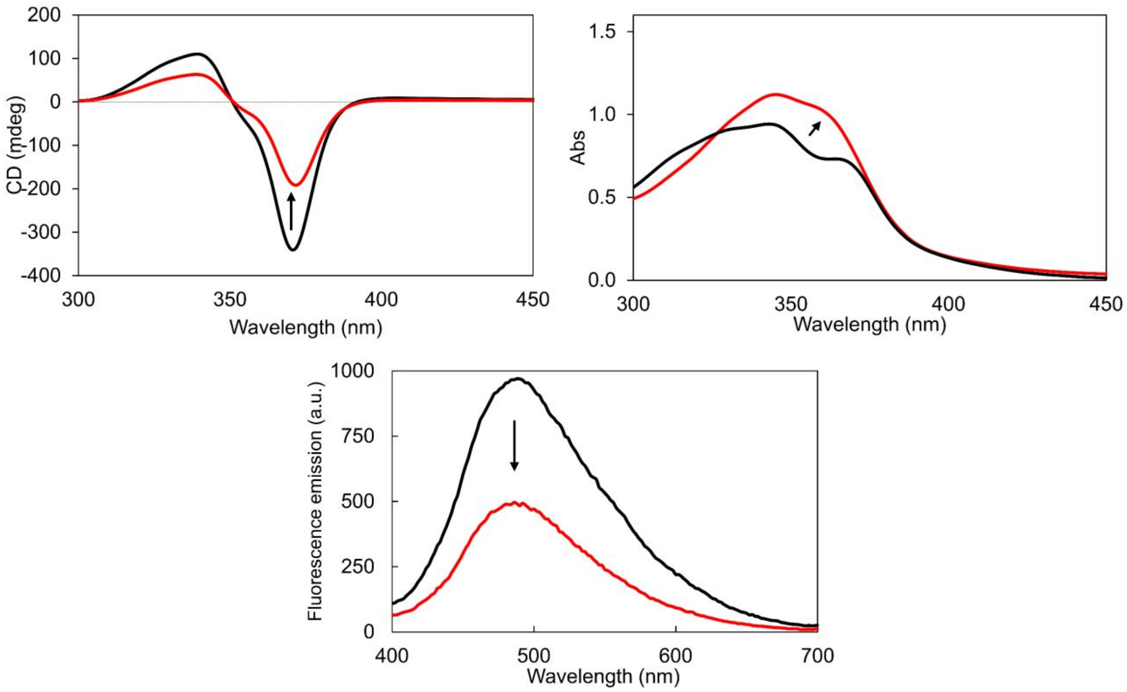

Figure S16. Non-normalized ECD (left), UV-vis (right) and emission (bottom) spectra of **1a**/***L*-TA** 1/125 aggregates in 15% (v/v) DMSO/H_2O before (black lines) and after (red lines) the addition of 2 equiv. of Ni^{2+}; spectra acquired after 3 days from metal addition; excitation slit = 20 nm, emission slit = 2.5 nm.

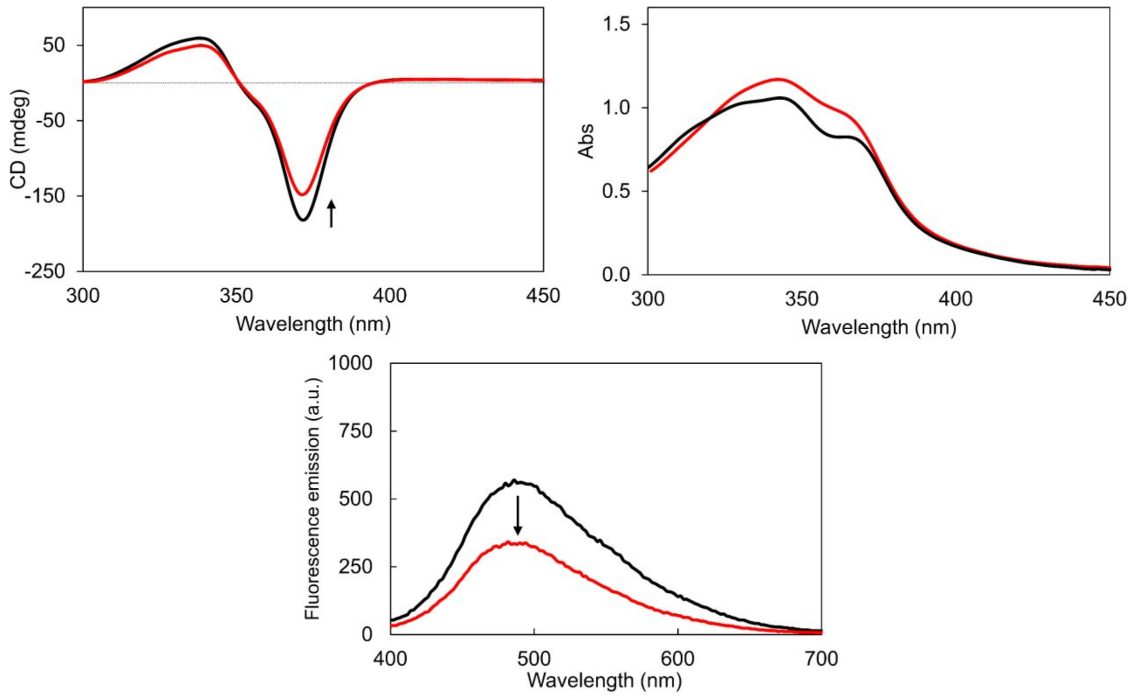

Figure S17. Non-normalized ECD (left), UV-vis (right) and emission (bottom) spectra of **1a/*L*-TA** 1/125 aggregates in 15% (v/v) DMSO/H_2O before (black lines) and after (red lines) the addition of 2 equiv. of Co^{2+}; spectra acquired after 3 days from metal addition; excitation slit = 10 nm, emission slit = 2.5 nm.

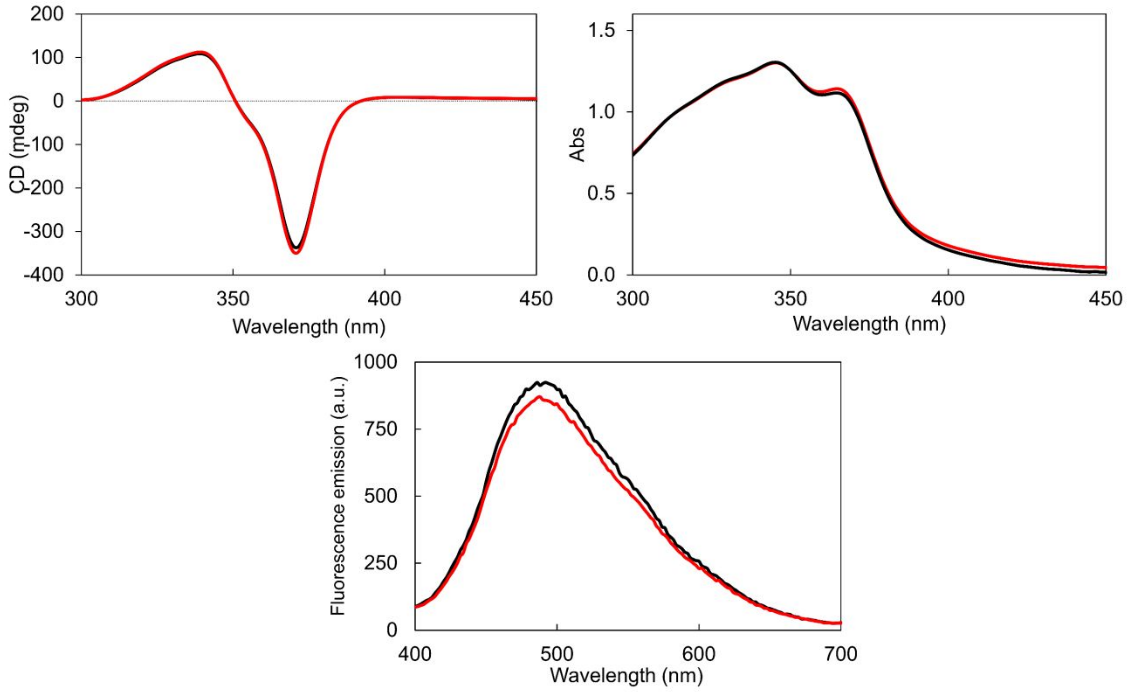

Figure S18. Non-normalized ECD (left), UV-vis (right) and emission (bottom) spectra of **1a/L-TA** 1/125 aggregates in 15% (v/v) DMSO/H_2O before (black lines) and after (red lines) the addition of 2 equiv. of Ba^{2+}; spectra acquired after 3 days from metal addition; excitation slit = 20 nm, emission slit = 2.5 nm.

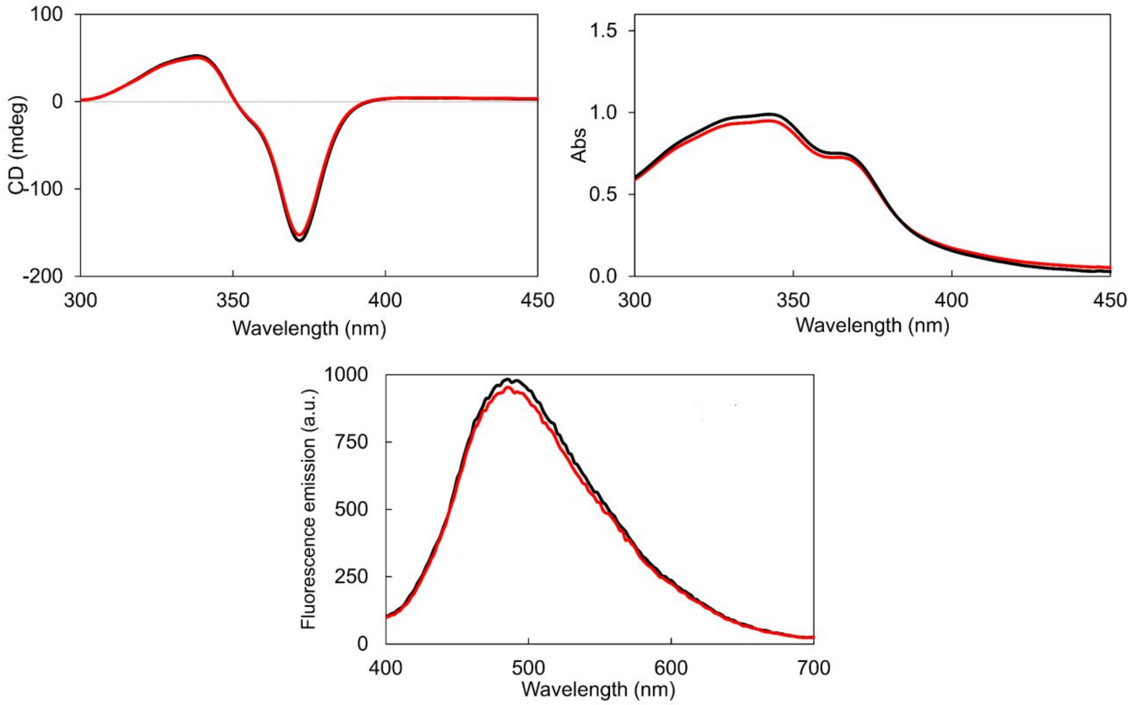

Figure S19. Non-normalized ECD (left), UV-vis (right) and emission (bottom) spectra of **1a**/***L*-TA** 1/125 aggregates in 15% (v/v) DMSO/H_2O before (black lines) and after (red lines) the addition of 2 equiv. of Eu^{3+}; spectra acquired after 3 days from metal addition; excitation slit = 20 nm, emission slit = 2.5 nm.

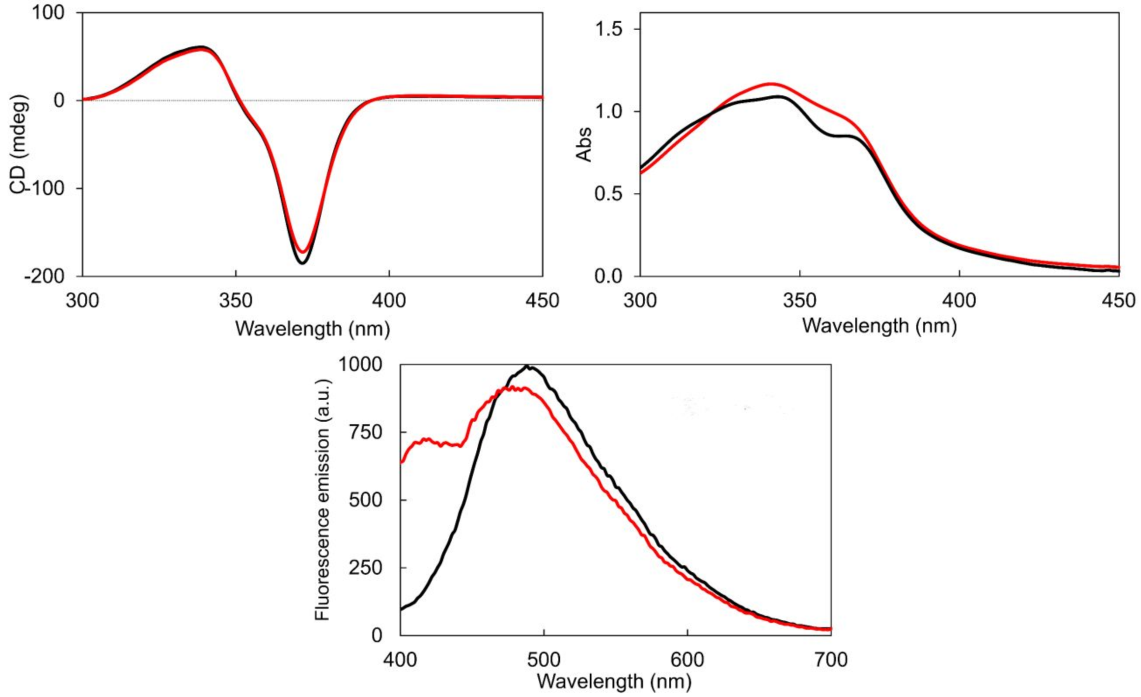

Figure S20. Non-normalized ECD (left), UV-vis (right) and emission (bottom) spectra of **1a**/***L*-TA** 1/125 aggregates in 15% (v/v) DMSO/H_2O before (black lines) and after (red lines) the addition of 2 equiv. of Zn^{2+}; spectra acquired after 3 days from metal addition; excitation slit = 20 nm, emission slit = 2.5 nm.

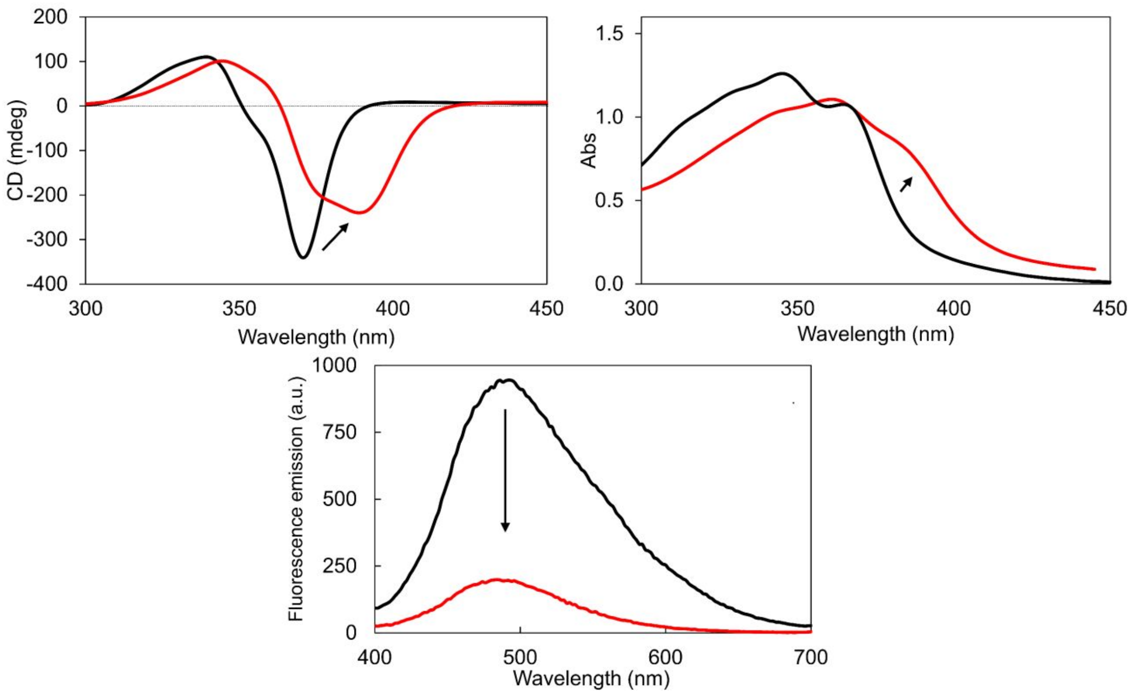

Figure S21. Non-normalized ECD (left), UV-vis (right) and emission (bottom) spectra of **1a**/***L*-TA** 1/125 aggregates in 15% (v/v) DMSO/H_2O before (black lines) and after (red lines) the addition of 2 equiv. of Cu^{2+}; spectra acquired after 3 days from metal addition; excitation slit = 20 nm, emission slit = 2.5 nm.

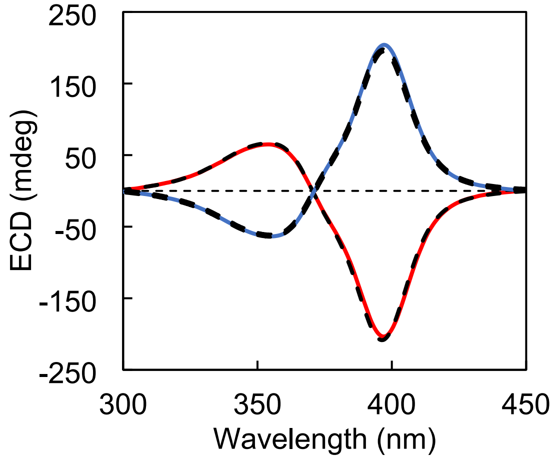

Figure S22. ECD spectra of ternary aggregates **1a**/**TA**/$\mathbf{Cu^{2+}}$ 1/125/100 in 15% (v/v) DMSO/H_2O before (solid blue (***D*-TA**) and red (***L*-TA**) lines) and 1 day after (dashed black lines) VT-ECD experiments heating at temperatures ranging from 20 to 90 °C and back to room temperature.

3. Additional References

S1) Preda, G.; Mobili, R.; Ravelli, D.; Amendola, V.; Pasini, D. Homoconjugation and Tautomeric Isomerism in Triptycene-Fused Pyridylbenzimidazoles. *J. Org. Chem.* **2024**, *89*, 5690–5698.

S2) Mobili, R.; Preda, G.; Dondi, D.; Monzani, E.; Vadivel, D.; Massera, C.; Pasini, D.; Amendola, V. Triptycene-based diiron(II) mesocates: spin-crossover in solution. *Chem. Commun.* **2024**, *60*, 5522-5525.

S3) Greenfield, N. J. Using Circular Dichroism Collected as a Function of Temperature to Determine the Thermodynamics of Protein Unfolding and Binding Interactions. *Nat. Protoc.* **2006**, *1*, 2527–2535.

S4) Huynh, K.; Partch, C. L. Analysis of Protein Stability and Ligand Interactions by Thermal Shift Assay. *Curr. Protoc. Protein Sci.* **2015**, *79*, 28.9.1-28.9.14.

S5) LeBel, R. G.; Goring, D. A. I. Density, Viscosity, Refractive Index, and Hygroscopicity of Mixtures of Water and Dimethyl Sulfoxide. *J. Chem. Eng. Data* **1962**, *7*, 100–101.

S6) Castiglioni, E.; Abbate, S.; Longhi, G. *Appl. Spectrosc.* **2010**, *64*, 1416–1419.

S7) Jo, S.; Kim, T.; Iyer, V. G.; Im, W. CHARMM-GUI: A Web-Based Graphical User Interface for CHARMM. *J. Comput. Chem.* **2008**, *29*, 1859–1865.

S8) Kim, S.; Lee, J.; Jo, S.; Brooks III, C. L.; Lee, H. S.; Im, W. CHARMM-GUI Ligand Reader and Modeler for CHARMM Force Field Generation of Small Molecules. *J. Comput. Chem.* **2017**, *38*, 1879–1886.

S9) Lee, J.; Cheng, X.; Swails, J. M.; Yeom, M. S.; Eastman, P. K.; Lemkul, J. A.; Wei, S.; Buckner, J.; Jeong, J. C.; Qi, Y.; Jo, S.; Pande, V. S.; Case, D. A.; Brooks, C. L. I.; MacKerell, A. D. Jr.; Klauda, J. B.; Im, W. CHARMM-GUI Input Generator for NAMD, GROMACS, AMBER, OpenMM, and CHARMM/OpenMM Simulations Using the CHARMM36 Additive Force Field. *J. Chem. Theory Comput.* **2016**, *12*, 405–413.

S10) Lee, J.; Hitzenberger, M.; Rieger, M.; Kern, N. R.; Zacharias, M.; Im, W. CHARMM-GUI Supports the Amber Force Fields. *J. Chem. Phys.* **2020**, *153*, 035103.

S11) Kern, N. R.; Lee, J.; Choi, Y. K.; Im, W. CHARMM-GUI Multicomponent Assembler for Modeling and Simulation of Complex Multicomponent Systems. bioRxiv September 1, 2023, p 2023.08.30.555590.

S12) Jorgensen, W. L.; Chandrasekhar, J.; Madura, J. D.; Impey, R. W.; Klein, M. L. Comparison of Simple Potential Functions for Simulating Liquid Water. *J. Chem. Phys.* **1983**, *79*, 926–935.

S13) Case, D. A.; Cheatham III, T. E.; Darden, T.; Gohlke, H.; Luo, R.; Merz Jr., K. M.; Onufriev, A.; Simmerling, C.; Wang, B.; Woods, R. J. The Amber Biomolecular Simulation Programs. *J. Comput. Chem.* **2005**, *26* (16), 1668–1688.

S14) *Self-Guided Langevin Dynamics (SGLD) simulation — CHARMM v35b1 documentation*. https://charmm-gui.org/charmmdoc/sgld.html (accessed 2024-01-17).

S15) Darden, T.; York, D.; Pedersen, L. Particle Mesh Ewald: An N·log(N) Method for Ewald Sums in Large Systems. *J. Chem. Phys.* **1993**, *98* (12), 10089–10092.

S16) Miyamoto, S.; Kollman, P. A. Settle: An Analytical Version of the SHAKE and RATTLE Algorithm for Rigid Water Models. *J. Comput. Chem.* **1992**, *13* (8), 952–962.
